# *MYT1L* haploinsufficiency in human neurons and mice causes autism-associated phenotypes that can be reversed by genetic and pharmacologic intervention

**DOI:** 10.1038/s41380-023-01959-7

**Published:** 2023-02-14

**Authors:** Bettina Weigel, Jana F. Tegethoff, Sarah D. Grieder, Bryce Lim, Bhuvaneswari Nagarajan, Yu-Chao Liu, Jule Truberg, Dimitris Papageorgiou, Juan M. Adrian-Segarra, Laura K. Schmidt, Janina Kaspar, Eric Poisel, Elisa Heinzelmann, Manu Saraswat, Marleen Christ, Christian Arnold, Ignacio L. Ibarra, Joaquin Campos, Jeroen Krijgsveld, Hannah Monyer, Judith B. Zaugg, Claudio Acuna, Moritz Mall

**Affiliations:** 1 Cell Fate Engineering and Disease Modeling Group, German Cancer Research Center (DKFZ) and DKFZ-ZMBH Alliance, 69120 Heidelberg, Germany; 2 HITBR Hector Institute for Translational Brain Research gGmbH, 69120 Heidelberg, Germany; 3 Central Institute of Mental Health, Medical Faculty Mannheim, Heidelberg University, 68159 Mannheim, Germany; 4 Faculty of Biosciences, Heidelberg University, 69120 Heidelberg, Germany; 5 Department of Clinical Neurobiology, University Hospital Heidelberg and DKFZ, Heidelberg, Germany; 6 Division of Proteomics of Stem Cells and Cancer, German Cancer Research Center (DKFZ), 69120 Heidelberg, Germany; 7 Medical Faculty, Heidelberg University, 69120 Heidelberg, Germany; 8 European Molecular Biology Laboratory, Structural and Computational Biology Unit, 69115 Heidelberg, Germany; 9 Chica and Heinz Schaller Research Group, Institute for Anatomy and Cell Biology, Heidelberg University, 69120 Heidelberg, Germany; 10 Present Address: Institute of Computational Biology, Helmholtz Zentrum München, German Research Center for Environmental Health, 85764 Neuherberg, Germany

**Keywords:** Neuroscience, Stem cells, Autism spectrum disorders

## Abstract

MYT1L is an autism spectrum disorder (ASD)-associated transcription factor that is expressed in virtually all neurons throughout life. How *MYT1L* mutations cause neurological phenotypes and whether they can be targeted remains enigmatic. Here, we examine the effects of MYT1L deficiency in human neurons and mice. Mutant mice exhibit neurodevelopmental delays with thinner cortices, behavioural phenotypes, and gene expression changes that resemble those of ASD patients. MYT1L target genes, including *WNT* and *NOTCH*, are activated upon MYT1L depletion and their chemical inhibition can rescue delayed neurogenesis in vitro. MYT1L deficiency also causes upregulation of the main cardiac sodium channel, *SCN5A*, and neuronal hyperactivity, which could be restored by shRNA-mediated knockdown of *SCN5A* or *MYT1L* overexpression in postmitotic neurons. Acute application of the sodium channel blocker, lamotrigine, also rescued electrophysiological defects in vitro and behaviour phenotypes in vivo. Hence, *MYT1L* mutation causes both developmental and postmitotic neurological defects. However, acute intervention can normalise resulting electrophysiological and behavioural phenotypes in adulthood.

## Introduction

Autism spectrum disorder (ASD) is a common neurodevelopmental disorder (NDD) characterised by behavioural changes, including altered social patterns [1]. ASD is often associated with coexisting conditions, including epilepsy, intellectual disability, and hyperactivity. Gene mutations affecting neuronal communication confer increased risk for ASD and offer possible therapeutic targets [2]. However, the genetic heterogeneity of ASD is enormous, and multiple transcriptional regulators have recently been associated with this group of disorders. Indeed, mouse models show that mutations of chromatin remodelers, such as *Chd8* and BAF complex members like *Smarcc2*, can induce behavioural phenotypes [3–6], suggesting a potential causal role in ASD. Yet, their contribution to disease and their clinical relevance often remains elusive [7], which limits the development of targeted treatments for gene regulator-associated mental disorders at the time of diagnosis [8, 9].

Of the 91 chromatin or gene regulators most strongly associated with ASD (category 1; [10]), MYT1L is specifically and continuously expressed in virtually all neurons throughout life [10–12]. MYT1L is a conserved zinc finger transcription factor and mutations have been reported in patients diagnosed with intellectual disability, schizophrenia, epilepsy, and ASD [13–18], suggesting that MYT1L-mediated gene regulation may be important in preventing NDDs including ASD. Indeed, 98% (50 out of 51) of currently reported cases with heterozygous *MYT1L* deletion or loss-of-function mutations were diagnosed with ASD and/or intellectual disability [19]. Besides behavioural features, several patients with *MYT1L* mutations also display developmental delays, obesity, seizures, and brain malformations [19]. MYT1L was one of the three original factors able to directly reprogram fibroblasts into functional neurons upon overexpression [20]. MYT1L is a transcriptional repressor that can enhance neuronal identity in vitro by actively repressing several developmental pathways, including WNT and NOTCH. This is achieved in part through recruitment of epigenetic silencers such as SIN3/HDAC [21–23]. Unexpectedly, reprogramming experiments have also revealed that MYT1L binds and represses several non-neuronal gene programs, such as muscle and fibroblast genes, suggesting a role as pan-neuronal safeguard that silences other lineage-specific genes [21, 24, 25]. Indeed, recent studies described that *Myt1l* haploinsufficiency, caused by frameshift mutation of *Myt1l* exon 15 or deletion of exon 9, induced altered brain development and behaviour phenotypes in mice [26, 27]. However, a number of important questions remain unanswered. For example, it is unclear whether distinct *MYT1L* mutations cause overlapping phenotypes, as suggested based on patient reports. Further, no studies present the effects of MYT1L depletion in human neurons. Finally, the molecular mechanisms causing the neurological phenotypes, and whether they are amenable to intervention, are unknown.

Here, we used genetically-engineered mice and human induced neurons to investigate the role of MYT1L during development and as a new preclinical platform to study MYT1L-associated NDDs. Using these translational models, we show that MYT1L deficiency is sufficient to induce autism-associated phenotypes, ranging from deregulation of gene expression and delayed neurogenesis to cortical thinning and altered behaviour. MYT1L target genes, including WNT and NOTCH regulators, were activated early upon MYT1L depletion in human neurons and chemical pathway inhibition could rescue associated differentiation defects. We found that MYT1L loss also caused upregulation of non-neuronal genes, including the cardiac sodium channel SCN5A, and triggered an unexpected neuronal network hyperactivity that can be rescued by *MYT1L* overexpression or *SCN5A* knockdown. Furthermore, acute application of the approved drug lamotrigine rescued the electrophysiological phenotypes in postmitotic mouse and human neurons, as well as behaviour phenotypes in adult mice, providing a potential therapeutic avenue for patients with MYT1L syndrome.

## Materials and methods

### Statistical analysis

No statistical methods were used to predetermine sample size. All values including replicates and statistical analysis are available in the Supplementary Tables S2–8, 13. Cells for in vitro analysis were randomly selected and mice were randomly allocated to treatment groups. The investigators were blinded for patch clamp recordings, other experiments were not randomised.

### Experimental design

To model MYT1L loss we generated a germline mutation in mice harbouring a 7 bp frameshift in *Myt1l* exon 6. In addition, we engineered a conditional heterozygous *MYT1L* allele in human ESCs, allowing cre-mediated removal of exon 7 in human neurons. We observed the expected reduction of full-length MYT1L protein in human and mouse neurons. In mutant mice we found a truncated MYT1L protein isoform that lacked nuclear localisation and function, confirming the validity of our loss-of-function models.

### MYT1L immunoprecipitation and mass spectrometry

One hemisphere of P0 mice was flash-frozen in liquid nitrogen and used for each MYT1L immunoprecipitation as described previously [21]. Bound proteins were enzymatically digested and peptides were analysed by mass spectrometric analysis (Fusion Orbitrap; Thermo Fisher Scientific).

### Primary tissue dissection and cell culture

Mouse glial cells were isolated from forebrains of wild-type CD1 (Jackson Laboratories) at P0 as described here [21]. For primary neuronal cultures, the hippocampus or the cortex of P0 *Myt1l*-mutant or control mouse pups were isolated as published previously [28]. For overexpression or knockdown, the cells were transduced with lentivirus encoding indicated constructs on day in vitro 3 (DIV3).

### Generation of human neurons

Human neuron generation has been described previously [29]. WNT and NOTCH inhibition was performed by supplementing the media from day 1 to day 4 with either 10 µM N-[N-(3,5-difluorophenacetyl)-l-alanyl]-S-phenylglycine t-butyl ester (DAPT; Sigma), 15 µM Tetrahydro-2-[4-(trifluoromethyl)phenyl]-4H-thiopyrano[4,3-d]pyrimidin (XAV939; Santa Cruz), or both. For functional maturation, neuronal cells were seeded on Matrigel-coated coverslips or PEI-laminin-coated MEA plates (Axion) together with primary mouse glia with media changes every 2 days for one week. Following media changes were performed every 3–4 days for the duration of the experiment.

### EdU labelling and immunostaining of mouse brain sections

Timed-pregnant females were intraperitoneally injected at E14.5 with EdU (30 mg/ kg body weight) (Life Technologies). After 20 h embryonic brains were harvested. Q-fraction analysis followed previously established practices [6]. The number of TBR2+ and SOX2+ cells was determined in midcortical 265 µm-wide segments using an in-house developed Fiji macro. All quantifications were carried out at equivalent anteroposterior positions between genotypes.

### Morphology analysis

For analysis of cortical thickness, P0 brain sections were aligned across genotypes using subcortical anatomical landmarks for orientation, and thickness was measured at a 45° angle from the dorsal midline. Morphology analysis of cultured neurons was performed with Fiji using SNT plugin [30, 31]. Nuclear FLAG-MYT1L localisation was performed in primary mouse neurons at DIV 11 upon overexpression of indicated constructs at DIV 3.

### Behaviour experiments

The mice were housed in a temperature-controlled vivarium maintained on a 12-h light–dark cycle and tests were conducted during the light cycle. Procedures were performed at the Interdisciplinary Neurobehavioral Core of Heidelberg University and approved by the Regierungspräsidium in Karlsruhe (G-287/20, G-105/16).

### Transcriptome analysis using RNA-sequencing

RNA-sequencing libraries were prepared following the dUTP protocol [32] and reads were mapped to hg38 or mm10 using STAR [33] and differential expression was determined using DESeq2 [34] (R package version 1.28.1) with size factor normalisation and Wald significance tests. For single-cell RNA-Seq experiments, prefrontal cortices from P0 mice were extracted, dissociated into single cells and barcoded following the ECCITE-Seq [35] scheme following a previously published protocol [36]. The Chromium Single Cell 5’ v1 reagent kit was employed for library preparation. Libraries were sequenced using a NovaSeq 6000 (Illumina). Data was analysed with 10x Genomics Cell Ranger (version 4.0.0) [37], Seurat (version 4.0) [38], and Scanpy (version 1.6.0) [39]. Subpopulations of each cell type most affected by *Myt1l* mutation were identified using MELD (version 1.0) [40] with parameters Beta = 31 and KNN = 5. Significant changes in cell type ratios were identified using a bootstrapping method [41] based on normalised cell numbers (FDR < 0.01 and abs(Log2FC) > 1). Differential expression was performed on these sub-populations using MAST (version 1.16.0) [42] within Seurat’s FindMarkers function (logfc.threshold = 0, min.pct = 0.05, all other parameters default). Sequencing reads are available on NCBI GEO GSE171327.

### Chromatin binding using CUT&RUN

The prefrontal cortex of E18.5, P0 and 3-months-old mice was prepped and single cells were prepared following the protocol for primary culture preparation. 300,000 cells per animal were used for CUT&RUN using published protocols [43]. Libraries were prepared with the NEBNext DNA Library Prep Kit and sequenced on NextSeq 2000 (Illumina). Data was analysed using the nf-core/cutandrun pipeline v1.0 (10.5281/zenodo.5653535). Homer findMotifsGenome.pl was run on the peak files with the parameters -size −75,75 -mask -mknown and the MYT1L motif AAAGTTW (http://homer.ucsd.edu/homer/).

### Brain slice preparation and electrophysiology

4–6 weeks-old-mice were used to generate and transverse hippocampal slices which were patched as described previously [44]. For spontaneous EPSCs measurements, pyramidal cells were voltage-clamped at −70 mV with a K^+^-based internal solution containing the following (in mM): 130 K-gluconate, 10 Na-gluconate, 10 HEPES, 10 phosphocreatine, 4 NaCl, 4 MgATP, 0.3 GTP and 0.5% biocytin. For spontaneous IPSCs measurements, putative pyramidal cells were voltage-clamped at 0 mV with a Cs^+^-based internal solution containing the following (in mM): 126 Cs-gluconate, 4 Cs-Cl, 10 HEPES, 10 phosphocreatine, 4 MgATP, 0.3 GTP and 2.5 QX-314 in the presence of CNQX (10 μM) and D-APV (50 μM). Recordings of excitatory synaptic currents in primary hippocampal cultures at DIV11 were performed as described here [21] in the presence or absence of 0.5 µM tetrodotoxin (TTX).

### Multi-electrode array (MEA) and drug treatment

MEA measurements were performed using the Maestro Pro multiwell device with the Axis Navigator software and 48-well plates (all from Axion Biosystems). For acute treatments, 10 µM lamotrigine (Targetmol) was added per well and the plate was measured before and 2 h after the treatment. Data was analysed using meaRtools [45].

## RESULTS

### MYT1L represses non-neuronal gene expression and is essential for survival in mice

**Fig. 1:**
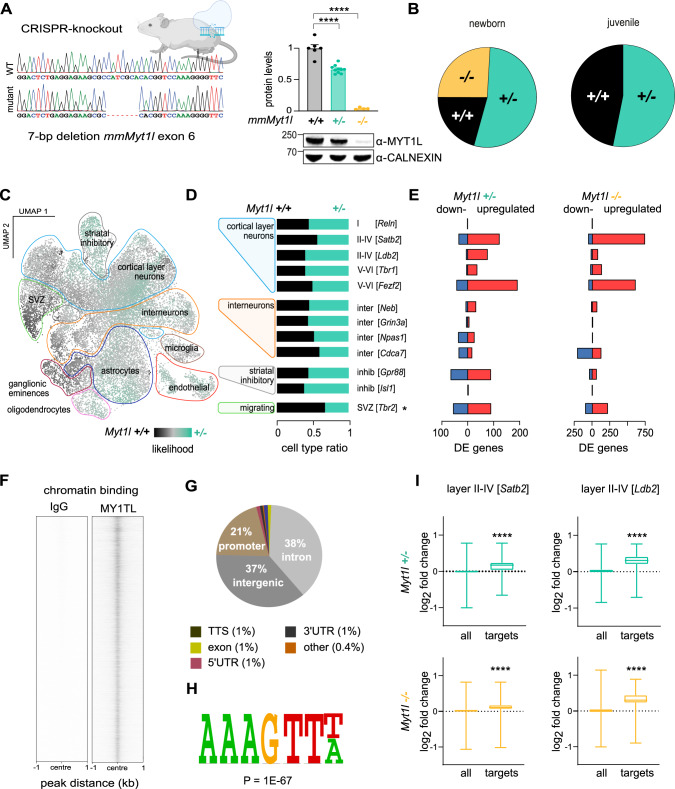
MYT1L is crucial for survival and repression of genes in mice. **A** MYT1L full-length protein levels in brain lysates from mice harbouring a CRISPR-induced 7 bp frameshift germline mutation in exon 6 (first protein coding exon) of *Myt1l* after birth normalised to control. Representative Western blot images using indicated antibodies are shown; *n* ≥ 6. **B** Survival pie chart from (+/−) x (+/−) offspring at birth (8 litters P0; *n* = 12 (+/+, black), 31 (+/−, teal), 15 (−/−, yellow)) and after weaning (7 litters P21; *n* = 15 (+/+), 17 (+/−), 0 (−/−)) indicated that homozygous *Myt1l* mutants die after birth. **C** Single cell RNA-Seq of the prefrontal cortex at birth and likelihood of observing MYT1L-deficient (*+/−*) and control (+/+) cell enrichment in populations of specific cell types using MELD. **D** Ratio of MYT1L-deficient (+/−) and control (+/+) cells in indicated cell types annotated based on reference data from [77]. * FDR < 0.01 & abs(Log2FC) > 1. **E** Number of genes deregulated upon *Myt1l* (+/−) and (−/−) mutation across indicated cell types and MELD clusters. Shown are genes that are down- (blue) or upregulated (red) upon MYT1L deficiency with absolute log2 fold change > 0.1 and *p*-adj < 0.05. **F** Genome-wide occupancy profiles of endogenous MYT1L in the prefrontal cortex of wildtype mice determined by CUT&RUN at P0 (*n* = 3). **G** Pie charts indicate the distribution of detected MYT1L-bound sites at annotated genomic regions. **H** The MYT1L DNA-binding motif (AAAGTT) is significantly enriched at bound sites. **I** Box plots of gene expression changes across the genome (all) and at MYT1L target genes (CUT&RUN peak ± 5 kb from transcription start site) for indicated single cell populations upon *Myt1l* deletion compared to control. For scRNA-Seq *n* = 2 for *Myt1l* (−/−; 10503 cells), (+/−; 13688 cells) and (+/+; 12072 cells), respectively. Bar graph shows mean values, data points from individual animals are displayed, error bars = SEM, unpaired *t*-test in panel A, Mann–Whitney test in panel I, ****p < 0.0001.

To address whether MYT1L mutations can cause complex neurodevelopmental defects, we generated a mouse model with a 7 bp deletion in exon 6 of *Myt1l* (Supplementary Fig. S1A–F). This frameshift mutation induces a premature STOP codon at amino acid (aa) 78, similar to a patient with a nonsense mutation at aa 75 in human MYT1L [15]. Resulting offspring exhibited expected reduction in full-length MYT1L protein compared to (+/+) controls (Fig. 1A and Supplementary Fig. S1G, H). However, we detected a truncated MYT1L isoform, most likely expressed from an internal methionine at aa 99 (Supplementary Fig. S1G, I) (Supplementary Table S1). This isoform failed to translocate to the nucleus due to lack of the nuclear localisation signal and therefore is expected to be non-functional (Supplementary Fig. S1J, K). *Myt1l* (+/−) mice are viable and fertile, but homozygous *Myt1l* (−/−) deletion resulted in postnatal lethality (Fig. 1B). This shows that MYT1L is essential for survival and validated our loss-of-function model.

To investigate changes in cell composition and gene expression, we performed single cell RNA sequencing (scRNA-Seq) of the prefrontal cortex at birth (Fig. 1C–E and Supplementary Fig. S2) (Supplementary Table S2). We did not observe loss or emergence of new cell clusters, but we identified *Myt1l* mutant-specific subpopulations by calculating the likelihood of each cell to be in a neighbourhood enriched in either control or mutant cells using MELD [40] (Fig. 1C and Supplementary Fig. S2C, D). Only minor changes in cell type ratios occurred between mutant and control mice (Fig. 1D and Supplementary Fig. S2E), but we found fewer *Tbr2*- and *Sema3c*-cell populations, which mark newly-formed migrating neurons in the subventricular zone (SVZ). Additionally, *Cdca7*+ interneurons were reduced in (−/−) mice, whereas cortical layer I neurons (*Reln*+ ) were increased. Analysis of the differentially expressed genes showed that MYT1L depletion resulted in global gene upregulation that was more pronounced in homozygous mutants (Fig. 1E). We observed increased expression of many non-neuronal gene programs upon MYT1L depletion, even in cortical layer neurons (Supplementary Fig. S2F). To identify MYT1L target genes, we performed CUT&RUN [43] in wildtype mice at embryonic (E) day E18.5, postnatal (P) day 0 (P0) and 3-months (adult) (Fig. 1F, G and Supplementary Fig. S2G, H) (Supplementary Table S3). We found a large overlap of bound sites and enrichment of the MYT1L DNA-binding motif (AAAGTT) at different developmental stages (Fig 1H and Supplementary Fig. S2I, J). Intersecting MYT1L target genes with deregulated genes at P0 revealed significant upregulation within different neuronal populations, such as *Satb2* or *Fezf2* cortical cell populations (Fig. 1I and Supplementary Fig. S2K). This underscores the role of MYT1L as a transcriptional repressor and suggests that, despite induction of neuronal identity, *Myt1l*-mutant cells failed to silence inappropriate gene expression programs causing a confused transcriptional identity in neurons in vivo.

### Impaired neurogenesis in *Myt1l*-mutant mice

**Fig. 2:**
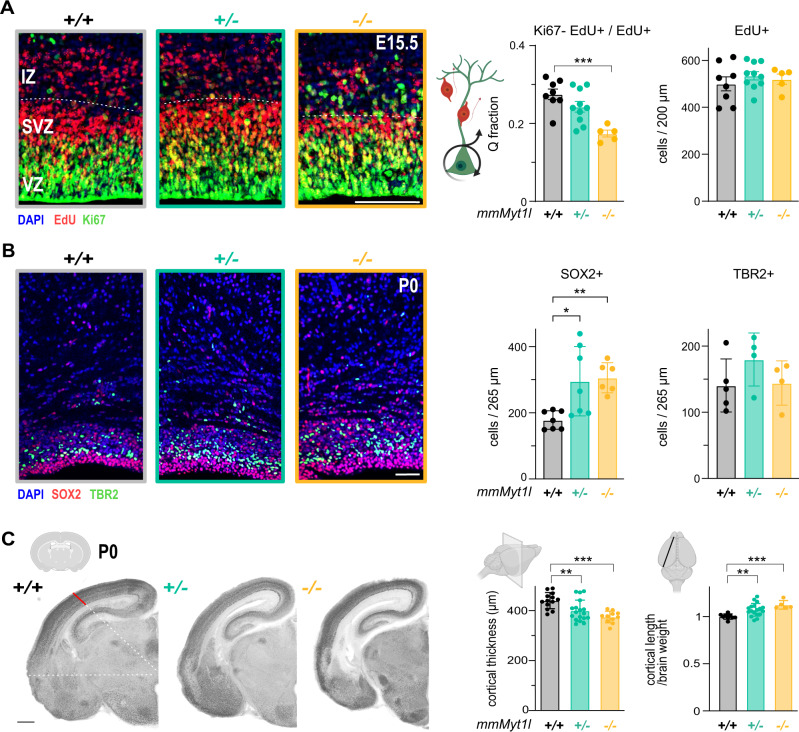
*Myt1l*-deficiency leads to impaired neurogenesis in mice. **A** Representative images and quantifications of EdU+ cells after a 20 h pulse at E14.5 and Q fraction (ratio of EdU+Ki67− over all EdU+ cells) in the cortical ventricular zone (VZ) and subventricular zone (SVZ) of *Myt1l* (+/+, black), (+/−, teal) and (−/−, yellow) E15.5 mice. IZ intermediate zone, *n* ≥ 5, scale bar 100 µm. **B** Quantification of SOX2+ or TBR2+ cells in the cortex of *Myt1l* (+/−) and (−/−) mice compared to (+/+) control at P0 across the same area of the entire cortex. Representative images of a ventricular and subventricular zone magnification stained with indicated antibodies are shown. *n* ≥ 4, scale bar 50 µm. **C** Structure of *Myt1l* (+/+), (+/−), and (+/−) brains at P0. Representative sections stained with NeuN and quantification of absolute cortical thickness and cortex length normalised by brain weight are shown. *n* ≥ 5 for cortical length; sections of *n* = 3 for cortical thickness, scale bar 500 µm. Bar graphs show mean values, data points from individual biological replicates are displayed, error bars = SEM, One-way ANOVA *p < 0.05,**p < 0.01, ***p < 0.001.

MYT1L can enhance neuronal identity in vitro by repressing negative regulators of neurogenesis [21], such as *Notch* and *Wnt*, which upon dysregulation could affect neuronal differentiation dynamics and brain structure. Therefore, we performed an EdU pulse-chase alongside co-staining for the proliferation marker, Ki67, to determine the quit (Q) fraction of cortical cells exiting the cell cycle over a 20 h period. Indeed, Ki67−EdU+ cells were significantly decreased, corresponding to a decrease in Q fraction of ~37% in *Myt1l* (-/-) embryos at E15.5 (Fig. 2A). SOX2+ neural stem cells and TBR2+ neural progenitors were unchanged at E15.5 (Supplementary Fig. 3A). At birth, SOX2+ stem cells were increased in *Myt1l* (+/−) and (−/−) mice (Fig. 2B), while TBR2+ progenitors did not change. This mimics overexpression of the NOTCH effector *Hes1* that increases the stem cell pool after birth and results in a thinner cortex [46]. Hence, we studied the brain anatomy of *Myt1l*-mutant mice and found that overall brain size was unchanged at birth while brain weight was lower, resulting in an increased length to weight ratio (Fig. 2C and Supplementary Fig. S3B, C). Furthermore, the cortices were ~10% (*+/*−) or ~15% (−/−) thinner than controls, respectively (Fig. 2C and Supplementary Fig. S3C). These results suggest that failure to repress anti-neurogenic programs impaired neurogenesis, which might in turn cause structural brain abnormalities and might explain brain malformations found in some patients [19].

### Gene expression changes in mutant mice resemble those of ASD patients

**Fig. 3:**
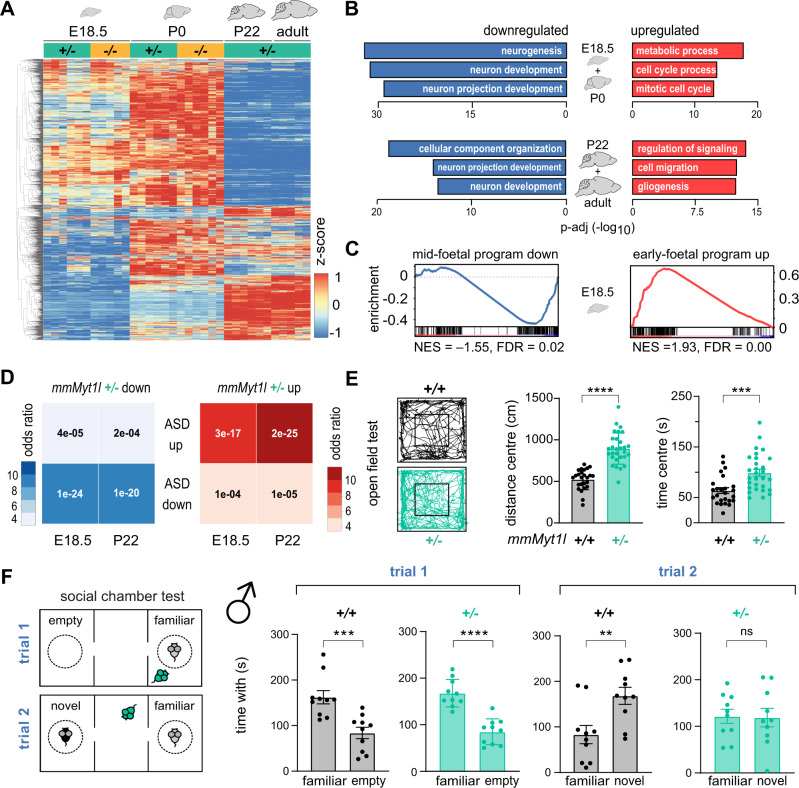
Autism-associated changes in gene expression and behavioural phenotypes upon *Myt1l* mutation in mice. **A** Heatmap of deregulated genes upon *Myt1l* mutation across all replicates and stages, scaled by gene (rows). **B** Selected top gene ontology (GO) terms and p-values of genes that were down- (blue) or upregulated (red) upon *Myt1l* mutation in the cortex of mice compared to control at indicated timepoints during development. **C** GSEA plots show depletion of mid-foetal (blue) and enrichment of early-foetal (red) neural development–related gene sets among genes deregulated in *Myt1l-*mutant brains at E18.5. NES, normalised enrichment score; FDR, false discovery rate. **D** Overlap of genes up- or downregulated upon *Myt1l* mutation in mouse cortex at E18.5 and P22 and genes that are up- or downregulated in ASD patient brains determined by GeneOverlap. For RNA-Seq analysis *n* ≥ 5 E18.5, *n* ≥ 4 P0 for (*+/+*, black), (*+/−*, teal) and (−/−, yellow), respectively; *n* = 6 P22, *n* ≥ 3 adult for (*+/+*) and (*+/−*), respectively. **E** Open field test showed that *Myt1l* (*+/−*) mutants covered more distance and spent more time in centre regions compared to control animals at P23; *n* = 25 for (*+/+*) and *n* = 29 for (*+/*−) from three independent cohorts. **F** Social chamber experiments showed male-specific decrease of time *Myt1l* (*+/*−) mutants spent to explore a novel mouse compared to control mice at the age of one month; *n* = 10 for (*+/+*) and (*+/−*) from three independent cohorts. Bar graphs show mean values, data points from individual animals are displayed, error bars = SEM, Mann–Whitney test **p < 0.01, ***p < 0.001, ****p < 0.0001, NS not significant.

Next, we examined the effects of MYT1L deficiency at the molecular level across brain development using bulk RNA-Seq of mouse cortices at E18.5 (coinciding with peak *Myt1l* expression in the mouse brain (Supplementary Fig. S1A [11])), P0 (birth), P22 (juvenile), and 3-months (adult). We found several hundred genes differentially expressed, with distinct clusters of deregulated genes early (E18.5 and P0) and late (P22 and adult) in development (Fig. 3A and Supplementary Fig. S4A) (Supplementary Table S4). Many of the deregulated genes did not overlap with previously-annotated MYT1L targets [21] or the MYT1L CUT&RUN targets (Fig. 1F and Supplementary Fig. S2G), implying that many changes in bulk samples are indirect. In heterozygous brain samples, MYT1L target genes were both up and downregulated, nevertheless ~60% of direct MYT1L target genes were upregulated upon homozygous *Myt1l* mutation (Supplementary Fig. S4B). This is in line with the upregulation of MYT1L target genes in (+/−) and (−/−) cortical neurons based on single cell analysis (Fig. 1E), suggesting that MYT1L predominantly acts as a repressor during neuronal development. In accordance with the decreased Q fraction at E15.5, gene expression at E18.5 revealed downregulation of neurogenesis-associated GO terms and upregulation of cell division (Fig. 3B). Furthermore, we observed upregulation of early-foetal gene expression signatures and concomitant decrease of late-foetal signatures at E18.5 [47] (Fig. 3C and Supplementary Fig. S4D). This suggests that MYT1L deficiency, as observed in other mouse models of ASD [5], causes delays in brain development. We observed continued downregulation of neuronal GO terms and an increase in signalling and non-neuronal terms upon *Myt1l* mutation in adult mice (Fig. 3B and Supplementary Fig. S4C). Gene set enrichment analysis (GSEA) highlighted several non-neuronal cell fate signatures among the upregulated genes, and indicated an overall enrichment of genes implicated in epilepsy, schizophrenia, and ASD among the deregulated genes in *Myt1l*-mutant brains [48, 49] (Supplementary Fig. S4D, E). Interestingly, mice harbouring our *Myt1l* exon 6 mutation displayed gene up- and downregulation that overlapped with *Chd8* haploinsufficient mice [6], but only partly with recently-published exon 15 [26] and exon 9 [27] *Myt1l*-mutant mice (Supplementary Fig. S4F). Finally, we discovered that genes upregulated in ASD patients are also upregulated in our MYT1L-deficient mice, while genes downregulated in *Myt1l*-mutant mice are also downregulated in ASD patients [5, 50] (Fig. 3D and Supplementary Fig. S4G). Overall, our data show that MYT1L deficiency in mice leads to delays in neurodevelopment and deregulation of gene expression as observed in ASD patients.

### Behavioural hyperactivity and male-specific social deficits upon *Myt1l* mutation in mice

Next, we performed behaviour analyses using our *Myt1l* mouse model. We observed a trend towards increased vocalisation in early postnatal MYT1L-deficient mice, but the number and type of analysed calls were not significantly changed (Supplementary Fig. S5A). In line with hyperactivity phenotypes reported in several MYT1L patients [13–16, 51, 52], we observed increased locomotion in adult mice in their home cages, as well as in juvenile mice in open field and elevated plus-maze tests (Fig. 3E and Supplementary Fig. S5B–D). Mutant mice spent ~70% more time in centre regions and open arms in the latter two tests, respectively. In addition, the explorative rearing behaviour was significantly increased in mutant mice (Supplementary Fig. S5D). This indicates that MYT1L-deficient mice are less anxious, a phenotype reported in other ASD mouse models [53, 54]. We also observed a significant decrease in marble burying and grooming events, which are used to evaluate repetitive behaviours but also represent decreased anxiety-like behaviours (Supplementary Fig. S5D, F). Finally, social chamber experiments were performed to investigate the social behaviour of our *Myt1l*-mutant mice. All animals displayed the expected preference for investigating chambers with a familiar littermate as opposed to empty chambers. However, while both wildtype and female *Myt1l*-mutant mice spent more time exploring the chamber with a newly-added unfamiliar mouse, MYT1L-deficient males did not exhibit this expected preference for social novelty (Fig. 3F and Supplementary Fig. S5E). In summary, *Myt1l* depletion causes several striking behavioural changes in tests used to model ASD-linked behaviour including hyperactivity that was found in male and female mice and male-specific social deficits.

### Delayed neurogenesis and non-neuronal gene expression in MYT1L-deficient human neurons

**Fig. 4:**
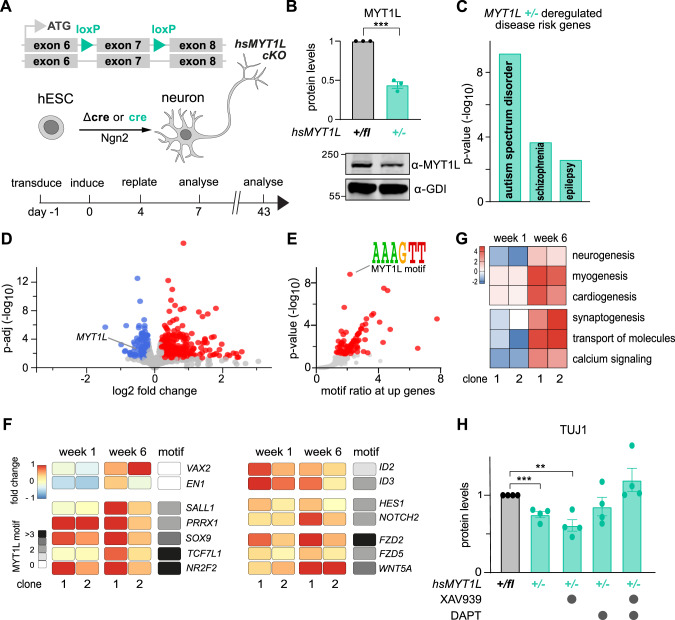
Heterozygous *MYT1L* deletion delays neurogenesis in a WNT- and NOTCH-dependent manner and causes deregulation of ASD-linked genes in human neurons. **A** Schematic of conditional heterozygous *MYT1L* deletion during transcription factor-mediated induced human neurogenesis. **B** Western blot quantification of cells 7 days (1 week) after induction of neurogenesis normalised to control. Representative Western blot images using indicated antibodies are shown; *n* = 3. **C** Deregulated genes in MYT1L-mutant neurons 43 days (6 weeks) after induction of neurogenesis normalised to control exhibited significant overlap with genes linked to epilepsy, schizophrenia, and ASD determined by Fisher’s exact test. **D** Volcano plot of differentially expressed genes in MYT1L-depleted human neurons after maturation for one week compared to isogenic controls. Highlighted are genes that were down- (blue) or upregulated (red) upon MYT1L depletion with absolute log2 fold change > 0.2 and *p*-adj < 0.1. **E** The MYT1L DNA binding motif AAAGTT was significantly enriched at upregulated genes in panel D. **F** Selected deregulated genes upon MYT1L depletion are displayed as fold change down- (blue) or upregulated (red) compared to isogenic control and number of MYT1L motifs in respective promoters is shown. B-E displays data for representative clone 1. **G** Ingenuity Pathway Analysis (IPA) of differentially-expressed genes in MYT1L-depleted human induced neurons. The state of activation (red) or inhibition (blue) of a pathway or biological function is represented by a z-score (right-tailed Fisher’s exact test). Results are displayed for two clones, 1 and 6 weeks after transcription factor-mediated induced human neurogenesis. *n* = 4 (clone 1) or *n* = 5 (clone 2) for (*+/fl*) and (+/−), respectively. **H** Reduced TUJ1 protein levels in MYT1L-depleted induced human neurons at day 7 can be restored by WNT and NOTCH inhibition via XAV939 and DAPT with *n* = 4 (clone 1). Bar graphs show mean values, data points from individual biological replicates are displayed, error bars = SEM, unpaired *t*-test in panel B and H, **p < 0.01, ***p < 0.001.

To dissect the role of MYT1L in human neurons we engineered human embryonic stem cells (hESCs) with a heterozygous conditional knockout allele of *MYT1L* exon 7 (*+/fl*) (Fig. 4A and Supplementary Fig. S6). *MYT1L* (+*/fl*) hESCs were directed towards electrically mature human neurons using NGN2 transcription factor-mediated differentiation [29]. This allows to study developmental and synaptic processes linked to neurological disorders in a conditional manner [55–57]. Cre-mediated *MYT1L* (+*/*−) deletion resulted in ~50% protein reduction, mimicking *MYT1L* haploinsufficiency found in patients (Fig. 4B, Supplementary Fig S6G). First, we assessed the transcriptional effects of MYT1L depletion compared to Δcre-transduced isogenic controls at early and late maturation timepoints (Supplementary Table S5). *MYT1L*-mutant neurons exhibited a decrease in neuronal GO terms and an enrichment of genes implicated in epilepsy, schizophrenia, and ASD among the deregulated genes [48, 49] (Fig. 4C and Supplementary Fig. S7A). GO terms associated with differentially upregulated genes strongly overlapped between our mouse and human models (Supplementary Fig. 7B) (Supplementary Table S6), indicating conserved effects. Overall, MYT1L depletion resulted in stronger gene activation early (week 1) and late (week 6) after neuronal induction (Fig. 4D and Supplementary Fig. S7C). The MYT1L DNA-binding motif (AAAGTT) was enriched at genes upregulated upon MYT1L depletion (Fig. 4E and Supplementary Fig. S7D, E). Several transcription factors regulating non-neuronal cell fates, such as mesoderm-specific *PRRX1* or glia switch regulator *SOX9* [58, 59], harbour MYT1L motifs and were upregulated in MYT1L-deficient neurons (Fig. 4F). GSEA revealed upregulation of non-neuronal gene expression programs, such as muscle cell fate, upon MYT1L depletion (Supplementary Fig. S7F). Ingenuity Pathway Analysis (IPA) confirmed that inappropriate developmental programs, such as myogenesis and cardiogenesis, were activated following *MYT1L* mutation, suggesting that they are continuously repressed by MYT1L (Fig. 4G and Supplementary Fig. S7G). On the other hand, neuronal transcription factors such as *VAX2* or *EN1* that do not contain MYT1L motifs in their promoter showed decreased expression after one week, but compared to controls exhibited an unexpected increased expression at week six (Fig. 4F). This expression pattern was mirrored in pathways involved in neuronal differentiation and function, including synaptogenesis and calcium signalling, that were initially repressed but later increased in MYT1L-deficient neurons (Fig. 4G and Supplementary Fig. S7G). Importantly, our findings regarding neuronal gene deregulation were recapitulated in neurons derived from an additional independently-engineered *MYT1L* (+*/fl*) hESC clone (Supplementary Fig. S6 and S7). Surprisingly, the delay in neuronal gene expression did not affect neuron complexity after ten days or six weeks of induced neurogenesis (Supplementary Fig. S8). During neuronal reprogramming of mouse fibroblasts MYT1L promoted neuronal fate by repressing anti-neurogenic genes such as *Id3* as well as WNT and NOTCH pathway members [21]. Here, we observed increased expression of these genes in MYT1L-deficient human neurons during neuronal induction (Fig. 4F). Combined application of chemical inhibitors targeting the WNT and NOTCH pathways restored TUJ1 protein to control levels and partially normalised gene expression changes in MYT1L mutants based on RNA-Seq measurements, particularly by decreasing expression of genes that are upregulated upon MYT1L depletion (Fig. 4H and Supplementary Fig. S9A, B) (Supplementary Table S7). Moreover, we identified several pro-neuronal transcription factors that normally peak early during induced neurogenesis and that were downregulated in MYT1L-mutant neurons at week one, but upregulated at week six (Supplementary Fig. S9C). Expression of these factors could be normalised in MYT1L-deficient neurons by WNT and NOTCH inhibition at week one (Supplementary Fig. S9D). Overall, this indicates that heterozygous deletion of *MYT1L* during human induced neurogenesis causes deregulation of autism-associated genes and activation of non-neuronal target genes that result in neurogenesis delays at least in part mediated by increased WNT and NOTCH signalling.

### Synaptic and network hyperactivity in *MYT1L-mutant* human and mouse neurons

**Fig. 5:**
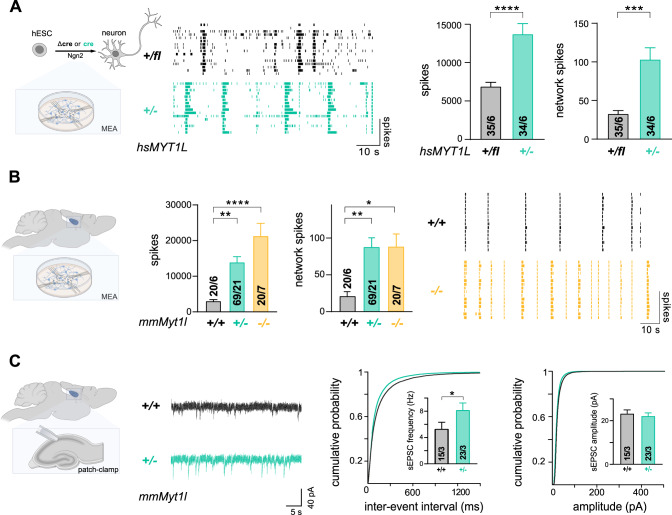
Electrophysiological hyperactivity in MYT1L-deficient human and mouse neurons in culture and brain slices. **A** Increased spontaneous neuronal network activity measured by multi-electrode array (MEA) in *MYT1L*-deficient (+/−; teal) neurons compared to control (+/fl, black) 6 weeks after induced neurogenesis. Representative raster plots and spike and network spike quantifications are shown. **B** Network hyperactivity measured by MEA for control (+/+, black) and mutant *Myt1l* (+/−; teal and −/−; yellow) hippocampal neurons in culture at day in vitro 11 (DIV11). Representative raster plots and quantifications are shown. **C** Increased spontaneous excitatory postsynaptic currents (sEPSCs) of CA1 pyramidal neurons in acute mouse brain slices. Representative traces, quantification and cumulative distributions are shown, bar graphs display mean values with number of patched cells or MEA wells from indicated biological replicates, error bars = SEM, Mann-Whitney–test in panel A and C, One-way ANOVA in panel B, *p < 0.05, ***p < 0.001, ****p < 0.0001.

To investigate whether *MYT1L* haploinsufficiency affects neuronal function in human cells, we studied the electrophysiological properties of *MYT1L* (+*/−*) neurons at the population level following six weeks of maturation using multi-electrode array (MEA) [60, 61]. Unexpectedly, we observed that spike firing doubled, and simultaneous network firing activity tripled, in human *MYT1L*-mutant neurons compared to controls (Fig. 5A). The altered electrophysiological activity directly correlated with MYT1L protein depletion, occurred as early as three weeks after neuronal induction and was maintained until the end of a long-term experiment at week 15 (Supplementary Fig. S10A–C). The electrophysiological phenotype could be recapitulated in an independently-engineered *MYT1L* (+*/fl*) hESC clone (Supplementary Fig. S10D). Next, we tested whether these functional changes were conserved in primary mouse neurons. To that end, we investigated electrophysiological properties of cultured hippocampal neurons derived from *Myt1l*-mutant and control mice using MEA. Recapitulating our findings in human *MYT1L* (+*/*−) neurons, we observed increased firing and network hyperactivity. These effects scaled with MYT1L depletion, with an average spike firing increase of ~700% (−/−) and ~400% (+/−) compared to control (Fig. 5B and Supplementary Fig. S11A). Of note, overexpression of MYT1L_99-1187_ in wildtype neurons did not alter electrophysiological activity on MEA, ruling out a potential dominant negative effect of the truncated MYT1L isoform further confirming that our *Myt1l*-mutant mice are a loss-of-function model (Supplementary Fig. S11B). Increased MEA firing activity could also be observed in primary neurons derived from cortices of MYT1L-deficient mice at birth, indicating that defects are not restricted to specific brain regions, but affected at least two areas implicated in ASD [62] (Supplementary Fig. S11C). To correlate the observed network hyperactivity with synaptic activity of single cells, we performed patch clamp recordings. In line with the observed network hyperactivity, the amplitude and frequency of spontaneous excitatory postsynaptic currents (EPSCs) and of miniature EPSCs (mEPSCs; recorded in the presence of tetrodotoxin (TTX)) was increased in MYT1L-deficient cultured primary mouse neurons (Supplementary Fig. S11D, E), respectively. Like in the human model, we did not observe significant changes in neuronal morphology of cultured MYT1L-deficient primary neurons (Supplementary Fig. S11F). We also performed electrophysiological brain slice recordings from pyramidal neurons located in the hippocampal CA1 region of one-month-old mice. Consistent with our in vitro data, we observed an increased spontaneous EPSC frequency in *Myt1l* mutants compared to controls (Fig. 5C). Intrinsic properties, such as resting membrane potential and evoked action potential features, appeared unchanged (Supplementary Fig. S12A). The frequency of spontaneous inhibitory postsynaptic currents (sIPSCs, recorded in the presence of CNQX and D-APV) was increased in MYT1L-deficient pyramidal neurons, indicating that the increased excitation did not result from decreased inhibition (Supplementary Fig. S12B). Our experiments show that continuous MYT1L depletion impairs neuronal function and results in an unexpected electrophysiological hyperactivity phenotype in human and mouse neurons.

### Genetic and pharmacologic rescue of neuronal network activity in vitro and behaviour phenotypes in vivo

**Fig. 6:**
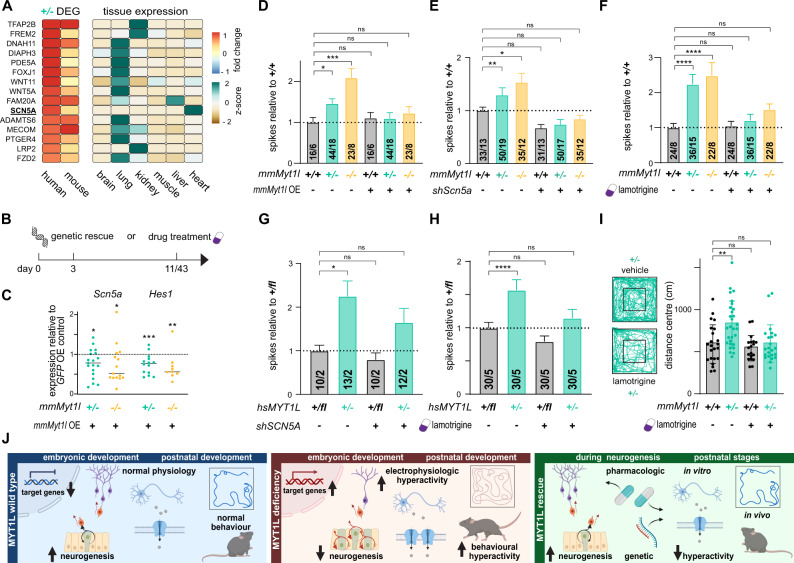
Electrophysiological and behaviour phenotypes caused by MYT1L deficiency can be normalised by genetic and pharmacological intervention. **A** Deregulated non-neuronal MYT1L target genes in *MYT1L*-deficient human neurons (week 6) and primary mouse neurons at day in vitro 11 (DIV11) include the cardiac sodium channel *SCN5A*. Deregulation of gene expression based on RNA-Seq is displayed as fold change compared to control, *n* = 4 (human) for (*+/fl*) and (*+/−*), *n* = 3 (mouse) for (+/+) and (+/-), respectively. Tissue expression data from GTEx portal is displayed as z-score. **B** Schematic timeline for the genetic and pharmacological rescue experiments in mouse primary hippocampal and induced human neuronal cultures. **C** Significant downregulation of MYT1L target genes *Scn5a* and *Hes1* in MYT1L-deficient mouse primary hippocampal cultures at DIV11 upon *Myt1l* overexpression (OE) at DIV3 compared to GFP OE determined by qRT-PCR. Graphs show column scatter plots with the median displayed; *n* ≥ 8. **D** Network hyperactivity in mouse primary neurons deficient of MYT1L can be rescued at DIV11 by overexpression of *Myt1l* in postmitotic neurons at DIV3. **E** Knockdown of *Scn5a* by shRNA expression in postmitotic neurons at DIV3 reduced spiking frequency of *Myt1l* (+/−) and (−/−) neurons. **F** Hyperactivity in mouse primary neurons deficient of MYT1L can also be rescued by acute application of 10 μM lamotrigine at DIV11. **G** *SCN5A* knockdown with shRNA in human induced neurons resulted in reduced spiking frequency. **H** Increased spike firing upon *MYT1L* mutation was normalised towards control by acute application of 10 μM lamotrigine in induced human neurons at week 6. **I** Open field tests showed that *Myt1l* (+/−) mutants covered more distance in centre regions compared to control animals at P23 and acute application of 20 mg/ kg lamotrigine can normalise these hyperactivity phenotypes. Vehicle: *n* = 23 for (*+/+*) and *n* = 29 for (*+/−*), lamotrigine: *n* = 19 for (*+/+*) and *n* = 23 for (*+/−*) data from three independent cohorts. **J** Schematic summary of the key roles of MYT1L during early and postnatal stages of development and defects upon MYT1L loss that can be compensated in part by genetic and pharmacologic intervention at both stages. Bar graphs display mean values with number of MEA wells from indicated biological replicates or data points from individual animals, error bars = SEM, Mann-Whitney test in panel C, Two-way ANOVA in panel D–I, *p < 0.05, **p < 0.01, ***p < 0.001, ****p < 0.0001, NS not significant.

We observed similar electrophysiological network hyperactivity phenotypes based on MEA measurements in primary mouse and stem cell-derived human induced neurons upon *MYT1L* mutation, indicating a conserved underlying molecular mechanism. Since activation of non-neuronal target genes upon *MYT1L* mutation was observed in human and mouse models, this might contribute to the overlapping phenotypes. To identify such targets, we analysed genes that: (i) were bound by MYT1L in vivo as determined by CUT&RUN (Fig. 1F and Supplementary Fig. S2G) (Supplementary Table S3); (ii) were deregulated in primary mouse or human induced neurons upon heterozygous *MYT1L* deletion determined by RNA-Seq (Supplementary Table S5 and 8); (iii) contributed to the upregulation of non-neuronal terms based on IPA (Fig. 4 and Supplementary Fig. S4 and 7); and (iv) were lowly expressed in the brain compared to other tissues, based on public expression datasets [63]. Strikingly, 78% (30 out of 38) of these non-neuronal targets were upregulated upon MYT1L depletion in mouse and human neurons (Fig. 6A and Supplementary Fig. S13A). The top upregulated target is *TFAP2B*, which, upon mutation, can cause Char syndrome, characterized by facial, ductal, and hand anomalies [64]. Interestingly, some patients with *MYT1L* mutations exhibit mild facial dysmorphisms [19, 65]. In addition, we found an almost two-fold increase of the cardiac voltage-gated sodium channel *SCN5A*, which upon mutation can cause long QT syndrome [66], among the top 10 upregulated target genes. *SCN5A* displayed a low basal expression in human and mouse neurons, and increased expression in MYT1L-deficient neurons could alter electrophysiological properties. *MYT1L* remains expressed in mature neurons, suggesting that it also functions in postmitotic neurons (Supplementary Fig. S1A). We therefore asked whether *Myt1l* overexpression (OE) in postmitotic MYT1L-deficient neurons could restore repression of target genes, such as *Scn5a*. *Myt1l* OE significantly increased the expression of the neuronal marker *Tuj1* compared to GFP OE controls (Fig. 6B and Supplementary Fig. S13B, C). Importantly, *Myt1l* OE decreased the expression of target genes that were upregulated in *MYT1L*-mutant neurons, specifically *Hes1* and *Scn5a* (Fig. 6C). Strikingly, we found that *Myt1l* OE also restored neuronal network activity to control levels (Fig. 6D). These results suggest that the continuous deregulation of gene expression in postmitotic neurons caused by *MYT1L* mutation contributes to electrophysiological hyperactivity phenotypes, and that these could be restored even after neurodevelopment is complete. To test whether *SCN5A* upregulation caused the electrophysiological hyperactivity phenotype, we performed shRNA-mediated *SCN5A* knockdown in both mouse primary and human induced neurons, which reduced *SCN5A* levels by 80%, and measured neuronal network activity using MEA (Supplementary Fig. S13D). Strikingly, *Scn5a* knockdown reduced neuronal network hyperactivity in (+/−) and (−/−) mouse primary neurons to wild type levels (Fig. 6E). This could be recapitulated by *SCN5A* knockdown in *MYT1L* (+/−) human induced neurons (Fig. 6G), indicating that *SCN5A* upregulation contributes at least in part to the electrophysiological network hyperactivity in mouse and human *MYT1L*-mutant neurons. In order to find a potential therapeutic intervention, we decided to test whether lamotrigine, a sodium channel blocker and FDA-approved antiepileptic drug [67, 68], could rescue MYT1L deficiency-induced phenotypes. Indeed, acute application of lamotrigine normalised electrophysiological network hyperactivity of both human and mouse MYT1L-deficient neurons towards control levels (Fig. 6F, H). Finally, to study if neuronal network hyperactivity contributes to the behaviour phenotypes observed upon *Myt1l* mutation and to test whether these are also amenable to pharmacological intervention in vivo, we injected mice with lamotrigine. We found that acute lamotrigine treatment in *Myt1l*-mutant mice normalised anxiety and hyperactivity behaviour in the open field test, as well as locomotion and rearing in home cage observations, towards untreated control mice (Fig. 6I and Supplementary Fig. S13E). Overall, these results show that, besides neurodevelopmental defects, *MYT1L* mutation causes upregulation of non-neuronal genes and neuronal network hyperactivity in mouse and human neurons. Acute lamotrigine treatment in post-mitotic neurons and adult mice rescued both the electrophysiological network hyperactivity and the tested behaviour phenotypes (Fig. 6J), indicating that electrophysiological effects contribute, at least in part, to altered behaviour in mice. Therefore, MYT1L mutation not only impacts development, but also affects neuronal function and behaviour in adulthood, suggesting that targeted treatments may also benefit patients at later stages of development.

## Discussion

Unlike most neuropsychiatric disease-associated chromatin regulators, the transcription factor MYT1L is specifically expressed in virtually all neurons and remains expressed throughout life [11, 12]. However, whether and how *MYT1L* mutations can cause neuropsychiatric disease remains poorly understood. In this study, we present evidence that *MYT1L* haploinsufficiency is sufficient to cause phenotypes associated with NDDs and ASD in human neurons as well as in mouse models due to failure to repress target genes resulting in (i) delays during developmental neurogenesis and (ii) synaptic malfunction in mature neurons. MYT1L deficiency in our human and mouse models caused significant deregulation of genes associated with epilepsy, schizophrenia, and ASD, which have been diagnosed in patients carrying *MYT1L* mutations [13–16]. We also found that gene expression changes in *Myt1l*-mutant mouse brains directly resembled those observed in ASD patient brains, demonstrating that MYT1L deficiency can lead to ASD-associated transcriptional profiles. Additional phenotypes linked to ASD and MYT1L-associated NDDs include neurodevelopmental delays and changes in brain anatomy. Using transcriptomic analysis, we observed altered maturation dynamics in MYT1L-deficient human neurons upon transcription factor-mediated neuronal differentiation in vitro and impaired neurogenesis in the prefrontal cortex of *Myt1l*-mutant mice in vivo. Even at E18.5, *Myt1l*-mutant mice displayed activation of early-foetal gene expression programs and downregulation of mid-foetal programs, which has also been described in other ASD models [5]. Previous studies showed that acute depletion of *Myt1l* or the closely related family member *Myt1* by shRNA treatment impaired neurogenesis in utero by repressing *Hes1* expression and Notch signalling [21, 69]. Here, we expand these findings by showing that germline *Myt1l* mutation impaired cell cycle exit of cortical progenitors during development and increased the number of SOX2+ neural stem cells in the SVZ at birth, which resembles *Hes1* overexpression phenotypes [46]. Indeed, combined chemical inhibition of WNT and NOTCH could restore early induction of proneuronal genes in human induced neurons. This confirms impaired neurogenesis upon MYT1L deficiency and could explain the thinned cortex observed in *Myt1l*-mutant mice and neurodevelopmental delays in human and mouse models.

One limitation of our study is that we modelled only frameshift mutations predicted to result in premature STOP codons of both human and mouse MYT1L, similar to a reported patient with a nonsense mutation at aa 75 [15]. Our mouse model presented a frameshift in exon 6 [70] that generated non-functional MYT1L protein isoforms missing essential domains such as the nuclear localisation signal, while conditional deletion of exon 7 in our human model resulted in nonsense-mediated RNA decay. Of note, both models exhibited similar gene deregulation and electrophysiology phenotypes, suggesting that loss-of-function results in overlapping defects independent of the mutation type. Notably, two recent studies describe additional *Myt1l* mouse models; Chen et al. generated a frameshift mutation in exon 15 [26], and Kim et al. used an exon 9 excision mutant [27]. All three models have in common that homozygous *Myt1l* knockout results in postnatal lethality and altered brain morphology, emphasising the crucial role of MYT1L for development. In addition, all three studies found behavioural phenotypes including hyperactivity. In line with our study, Kim et al. reported decreased anxiety. Chen et al. reported male-specific impaired social behaviour, which we also found in our model although using different assays and developmental stages. Nevertheless, the three *Myt1l*-mutant mouse models exhibit distinct gene expression and neurodevelopmental defects. In contrast to our study, Chen et al. report an increased Q fraction and a decrease in SOX2+ neural stem cells in *Myt1l*-deficient mice at E14.5 and suggested MYT1L mainly acts as a transcriptional activator. However, in a recent preprint the same authors found that MYT1L bound and repressed promoters and enhancers of genes involved in early neuronal development programs [71]. Here, using single cell transcriptomics, we show that MYT1L-bound genes are indeed activated in cortical neurons upon loss of MYT1L, supporting its role as a repressor. In line with this, both Chen et al. and our study found increased expression of early neurodevelopmental gene signatures later in development. Notably, all studies report a significant overlap of deregulated genes with ASD gene sets. Hence, while the differences between these three *Myt1l* mouse models might reflect the distinct nature of mutations, genetic mouse background, and experimental conditions, their overlap emphasises the important role of MYTL for neurodevelopment. In addition to loss-of-function variants, several de novo missense variants, indels and genomic duplications and deletions encompassing *MYT1L* have been reported in individuals with neuropsychiatric disease [19, 65]. Since MYT1L patients display diverse phenotypes, including both macro- and microcephaly, and are diagnosed with different neuropsychiatric disorders like ASD and schizophrenia, future studies will be needed to clarify how specific mutations or genetic backgrounds affect these phenotypes by engineering additional mouse and human stem cell models or by generating patient-derived neurons from induced pluripotent stem cells.

Alongside destabilization of neuronal cell identity, which manifested as gene expression changes and neurogenesis delays, we observed striking electrophysiological phenotypes in MYT1L-deficient neurons. Unexpectedly, primary mouse and induced human neurons displayed a three to four-fold increase in neuronal network activity, underpinned by an increase in sEPSC amplitude and mEPSC frequency. Since, the frequency of sIPSCs was increased in MYT1L-deficient pyramidal neurons, increased network activity is not likely caused by decreased inhibition in our model. However, in newborn *Myt1l* (−/−) mice, we observed a slight increase in the fraction of cortical layer I neurons along with decreased *Cdca7*+ interneuron populations based on single cell analysis. Therefore, the effect of MYT1L mutations on inhibitory neurons remains elusive and requires future studies. Network phenotypes could be explained by multiple factors, such as changes in neuronal morphology or synapse density as well as general mechanisms regulating neurotransmitter release, including deregulation of voltage-gated sodium and calcium channels [72–74]. Interestingly, the upregulation of channels that are normally not specifically expressed in neurons, such as *SCN5A*, could also impair normal synaptic transmission in MYT1L-mutant neurons. We therefore tested whether MYT1L overexpression, shRNA-mediated *SCN5A* knockdown or lamotrigine treatment, which is reported to block sodium and calcium channels [67, 68], could rescue the *MYT1L* mutation-induced network hyperactivity. Strikingly, MYT1L overexpression decreased expression of target genes such as *Scn5a* and *Hes1* in postmitotic MYT1L-deficient mouse neurons, and MYT1L overexpression and *SCN5A* knockdown both rescued electrophysiological network hyperactivity phenotypes. In addition, acute application of lamotrigine normalised not only the network hyperactivity phenotypes of post-mitotic MYT1L-deficient human and mouse neurons, but also several behavioural hyperactivity phenotypes observed in mice. This suggests that specific MYT1L haploinsufficiency-associated phenotypes can be rescued by genetic intervention or small-molecule drugs even later in development.

Overall, we present the first evidence that MYT1L mutations destabilise neuronal cell fate and function, and are sufficient to cause ASD-associated phenotypes in human and mouse models. Hence, failure to silence non-neuronal gene expression in neurons represents a novel mechanism that, at least in part, could contribute to ASD aetiology. MYT1L is a unique neurodevelopmental disease-associated transcription factor that is specifically expressed in virtually all neurons throughout life. It is therefore tempting to speculate that active lifelong repression of non-neuronal programs is an evolutionarily-conserved pathway critical for the prevention of brain disorders. Interestingly, *MYT1L* levels decrease during aging in both mice and humans (Supplementary Fig. S1A), and loss of neuronal cell identity has recently been suggested to play a role in Alzheimer’s disease models [75, 76], which suggests that neurodegeneration could also be regulated by the novel MYT1L-mediated mechanism presented here. Finally, we show that unexpected electrophysiological network hyperactivity upon MYT1L deficiency in post-mitotic mouse and human neurons, and associated behaviour phenotypes in mice, can be acutely targeted by the approved drug lamotrigine, which could provide an opportunity for therapeutic interventions.

### Supplementary information

Supplementary text

Supplementary Figure 1

Supplementary Figure 2

Supplementary Figure 3

Supplementary Figure 4

Supplementary Figure 5

Supplementary Figure 6

Supplementary Figure 7

Supplementary Figure 8

Supplementary Figure 9

Supplementary Figure 10

Supplementary Figure 11

Supplementary Figure 12

Supplementary Figure 13

Supplementary Table 1

Supplementary Table 2

Supplementary Table 3

Supplementary Table 4

Supplementary Table 5

Supplementary Table 6

Supplementary Table 7

Supplementary Table 8

Supplementary Table 9

Supplementary Table 10

Supplementary Table 11

Supplementary Table 12

Supplementary Table 13

## Data Availability

All data are available in the main text or the supplementary materials. GSEA was performed as described above. Next-generation sequencing data is available on NCBI GEO GSE171327. Mass spectrometry data can be accessed on PRIDE PXD037867.

## References

[1] Lord C, Elsabbagh M, Baird G, Veenstra-Vanderweele J (2018). Autism spectrum disorder. Lancet.

[2] Bourgeron T (2015). From the genetic architecture to synaptic plasticity in autism spectrum disorder. Nat Rev Neurosci.

[3] Tuoc T, Dere E, Radyushkin K, Pham L, Nguyen H, Tonchev AB (2017). Ablation of BAF170 in developing and postnatal dentate gyrus affects neural stem cell proliferation, differentiation, and learning. Mol Neurobiol.

[4] Tuoc TC, Boretius S, Sansom SN, Pitulescu M-E, Frahm J, Livesey FJ (2013). Chromatin regulation by BAF170 controls cerebral cortical size and thickness. Dev Cell.

[5] Katayama Y, Nishiyama M, Shoji H, Ohkawa Y, Kawamura A, Sato T (2016). CHD8 haploinsufficiency results in autistic-like phenotypes in mice. Nature.

[6] Gompers AL, Su-Feher L, Ellegood J, Copping NA, Riyadh MA, Stradleigh TW (2017). Germline Chd8 haploinsufficiency alters brain development in mouse. Nat Neurosci.

[7] Schaaf CP, Betancur C, Yuen RKC, Parr JR, Skuse DH, Gallagher L (2020). A framework for an evidence-based gene list relevant to autism spectrum disorder. Nat Rev Genet.

[8] Satterstrom FK, Kosmicki JA, Wang J, Breen MS, Rubeis SD, An JY (2020). Large-scale exome sequencing study implicates both developmental and functional changes in the neurobiology of autism. Cell.

[9] Turner TN, Coe BP, Dickel DE, Hoekzema K, Nelson BJ, Zody MC (2017). Genomic patterns of de novo mutation in simplex autism. Cell.

[10] Simons Foundation Autism Research Initiative SFARI. Highest ranking candidate autism risk genes. December 2019. https://www.sfari.org/resource/sfari-gene.

[11] Cardoso-Moreira M, Halbert J, Valloton D, Velten B, Chen C, Shao Y (2019). Gene expression across mammalian organ development. Nature.

[12] Matsushita F, Kameyama T, Kadokawa Y, Marunouchi T (2014). Spatiotemporal expression pattern of Myt/NZF family zinc finger transcription factors during mouse nervous system development. Dev Dynam.

[13] Lee Y, Mattai A, Long R, Rapoport JL, Gogtay N, Addington AM (2012). Microduplications disrupting the MYT1L gene (2p25.3) are associated with schizophrenia. Psychiatr Genet.

[14] Rocker ND, Vergult S, Koolen D, Jacobs E, Hoischen A, Zeesman S (2015). Refinement of the critical 2p25.3 deletion region: the role of MYT1L in intellectual disability and obesity. Genet Med.

[15] Windheuser IC, Becker J, Cremer K, Hundertmark H, Yates LM, Mangold E (2020). Nine newly identified individuals refine the phenotype associated with MYT1L mutations. Am J Med Genet Part A.

[16] Blanchet P, Bebin M, Bruet S, Cooper GM, Thompson ML, Duban-Bedu B (2017). MYT1L mutations cause intellectual disability and variable obesity by dysregulating gene expression and development of the neuroendocrine hypothalamus. Plos Genet.

[17] Kim JG, Armstrong RC, Agoston DV, Robinsky A, Wiese C, Nagle J (1997). Myelin transcription factor 1 (Myt1) of the oligodendrocyte lineage, along with a closely related CCHC zinc finger, is expressed in developing neurons in the mammalian central nervous system. J Neurosci Res.

[18] Weiner JA, Chun J (1997). Png‐1, a nervous system‐specific zinc finger gene, identifies regions containing postmitotic neurons during mammalian embryonic development. J Comp Neurol.

[19] Mansfield P, Constantino JN, Baldridge D (2020). MYT1L: a systematic review of genetic variation encompassing schizophrenia and autism. Am J Med Genet Part B Neuropsychiatr Genet.

[20] Vierbuchen T, Ostermeier A, Pang ZP, Kokubu Y, Sudhof TC, Wernig M (2010). Direct conversion of fibroblasts to functional neurons by defined factors. Nature.

[21] Mall M, Kareta MS, Chanda S, Ahlenius H, Perotti N, Zhou B (2017). Myt1l safeguards neuronal identity by actively repressing many non-neuronal fates. Nature.

[22] Manukyan A, Kowalczyk I, Melhuish TA, Lemiesz A, Wotton D (2018). Analysis of transcriptional activity by the Myt1 and Myt1l transcription factors. J Cell Biochem.

[23] Romm E, Kim JG, Kim NW, Nagle J, Hudson LD (2002). The MyT1 family recruits histone deacetylase to regulate neural transcription. J Neurochem.

[24] Treutlein B, Lee QY, Camp JG, Mall M, Koh W, Shariati SAM (2016). Dissecting direct reprogramming from fibroblast to neuron using single-cell RNA-seq. Nature.

[25] Lee QY, Mall M, Chanda S, Zhou B, Sharma KS, Schaukowitch K (2020). Pro-neuronal activity of Myod1 due to promiscuous binding to neuronal genes. Nat Cell Biol.

[26] Chen J, Lambo ME, Ge X, Dearborn JT, Liu Y, McCullough KB (2021). A MYT1L syndrome mouse model recapitulates patient phenotypes and reveals altered brain development due to disrupted neuronal maturation. Neuron.

[27] Kim S, Oh H, Choi SH, Yoo Y-E, Noh YW, Cho Y (2022). Postnatal age-differential ASD-like transcriptomic, synaptic, and behavioral deficits in Myt1l-mutant mice. Cell Rep.

[28] Maximov A, Pang ZP, Tervo DGR, Sudhof TC (2007). Monitoring synaptic transmission in primary neuronal cultures using local extracellular stimulation. J Neurosci Meth.

[29] Zhang Y, Pak CH, Han Y, Ahlenius H, Zhang Z, Chanda S (2013). Rapid single-step induction of functional neurons from human pluripotent stem cells. Neuron.

[30] Schindelin J, Arganda-Carreras I, Frise E, Kaynig V, Longair M, Pietzsch T (2012). Fiji: an open-source platform for biological-image analysis. Nat Methods.

[31] Arshadi C, Günther U, Eddison M, Harrington KIS, Ferreira TA (2021). SNT: a unifying toolbox for quantification of neuronal anatomy. Nat Methods.

[32] Levin JZ, Yassour M, Adiconis X, Nusbaum C, Thompson DA, Friedman N (2010). Comprehensive comparative analysis of strand-specific RNA sequencing methods. Nat Methods.

[33] Dobin A, Davis CA, Schlesinger F, Drenkow J, Zaleski C, Jha S (2013). STAR: ultrafast universal RNA-seq aligner. Bioinformatics.

[34] Love MI, Huber W, Anders S (2014). Moderated estimation of fold change and dispersion for RNA-seq data with DESeq2. Genome Biol.

[35] Mimitou EP, Cheng A, Montalbano A, Hao S, Stoeckius M, Legut M (2019). Multiplexed detection of proteins, transcriptomes, clonotypes and CRISPR perturbations in single cells. Nat Methods.

[36] Gehring J, Park JH, Chen S, Thomson M, Pachter L (2020). Highly multiplexed single-cell RNA-seq by DNA oligonucleotide tagging of cellular proteins. Nat Biotechnol.

[37] Zheng GXY, Terry JM, Belgrader P, Ryvkin P, Bent ZW, Wilson R (2017). Massively parallel digital transcriptional profiling of single cells. Nat Commun.

[38] Hao Y, Hao S, Andersen-Nissen E, Mauck WM, Zheng S, Butler A, et al. Integrated analysis of multimodal single-cell data. Cell. 2021;183:3573–87.e29. 10.1016/j.cell.2021.04.048 PMC8238499 34062119

[39] Wolf FA, Angerer P, Theis FJ (2018). SCANPY: Large-scale single-cell gene expression data analysis. Genome Biol.

[40] Burkhardt DB, Stanley JS, Tong A, Perdigoto AL, Gigante SA, Herold KC, et al. Quantifying the effect of experimental perturbations at single-cell resolution. Nat Biotechnol. 2021;39:619–29. 10.1038/s41587-020-00803-5 PMC8122059 33558698

[41] Miller SA, Policastro RA, Sriramkumar S, Lai T, Huntington TD, Ladaika CA (2021). LSD1 and aberrant DNA methylation mediate persistence of enteroendocrine progenitors That Support BRAF-mutant colorectal cancer. Cancer Res.

[42] Finak G, McDavid A, Yajima M, Deng J, Gersuk V, Shalek AK (2015). MAST: a flexible statistical framework for assessing transcriptional changes and characterizing heterogeneity in single-cell RNA sequencing data. Genome Biol.

[43] Skene PJ, Henikoff JG, Henikoff S (2018). Targeted in situ genome-wide profiling with high efficiency for low cell numbers. Nat Protoc.

[44] Liu Y-C, Cheng J-K, Lien C-C (2014). Rapid dynamic changes of dendritic inhibition in the dentate gyrus by presynaptic activity patterns. J Neurosci.

[45] Gelfman S, Wang Q, Lu Y-F, Hall D, Bostick CD, Dhindsa R (2018). MeaRtools: an R package for the analysis of neuronal networks recorded on microelectrode arrays. Plos Comput Biol.

[46] Ohtsuka T, Kageyama R (2021). Hes1 overexpression leads to expansion of embryonic neural stem cell pool and stem cell reservoir in the postnatal brain. Development.

[47] Kang HJ, Kawasawa YI, Cheng F, Zhu Y, Xu X, Li M (2011). Spatio-temporal transcriptome of the human brain. Nature.

[48] Ran X, Li J, Shao Q, Chen H, Lin Z, Sun ZS (2015). EpilepsyGene: a genetic resource for genes and mutations related to epilepsy. Nucleic Acids Res.

[49] Hormozdiari F, Penn O, Borenstein E, Eichler EE (2015). The discovery of integrated gene networks for autism and related disorders. Genome Res.

[50] Voineagu I, Wang X, Johnston P, Lowe JK, Tian Y, Horvath S (2011). Transcriptomic analysis of autistic brain reveals convergent molecular pathology. Nature.

[51] Tuwaijri AA, Alfadhel M (2019). MYT1L mutation in a patient causes intellectual disability and early onset of obesity: a case report and review of the literature. J Pediatr Endocrinol Metab.

[52] Loid P, Mäkitie R, Costantini A, Viljakainen H, Pekkinen M, Mäkitie O (2018). A novel MYT1L mutation in a patient with severe early‐onset obesity and intellectual disability. Am J Med Genet A.

[53] Eadie BD, Zhang WN, Boehme F, Gil-Mohapel J, Kainer L, Simpson JM (2009). Fmr1 knockout mice show reduced anxiety and alterations in neurogenesis that are specific to the ventral dentate gyrus. Neurobiol Dis.

[54] Samaco RC, Fryer JD, Ren J, Fyffe S, Chao H-T, Sun Y (2008). A partial loss of function allele of Methyl-CpG-binding protein 2 predicts a human neurodevelopmental syndrome. Hum Mol Genet.

[55] Yi F, Danko T, Botelho SC, Patzke C, Pak C, Wernig M (2016). Autism-associated SHANK3 haploinsufficiency causes Ih channelopathy in human neurons. Science.

[56] Chanda S, Ang CE, Lee QY, Ghebrial M, Haag D, Shibuya Y (2019). Direct reprogramming of human neurons identifies MARCKSL1 as a pathogenic mediator of valproic acid-induced teratogenicity. Cell Stem Cell.

[57] Pak C, Danko T, Zhang Y, Aoto J, Anderson G, Maxeiner S (2015). Human neuropsychiatric disease modeling using conditional deletion reveals synaptic transmission defects caused by heterozygous mutations in NRXN1. Cell Stem Cell.

[58] Martin JF, Bradley A, Olson EN (1995). The paired-like homeo box gene MHox is required for early events of skeletogenesis in multiple lineages. Gene Dev.

[59] Stolt CC, Lommes P, Sock E, Chaboissier M-C, Schedl A, Wegner M (2003). The Sox9 transcription factor determines glial fate choice in the developing spinal cord. Gene Dev.

[60] Flaherty E, Zhu S, Barretto N, Cheng E, Deans PJM, Fernando MB (2019). Neuronal impact of patient-specific aberrant NRXN1α splicing. Nat Genet.

[61] Liu XS, Wu H, Krzisch M, Wu X, Graef J, Muffat J (2018). Rescue of fragile X syndrome neurons by DNA methylation editing of the FMR1 gene. Cell.

[62] Amaral DG, Schumann CM, Nordahl CW (2008). Neuroanatomy of autism. Trends Neurosci.

[63] Consortium TGte. (2020). The GTEx Consortium atlas of genetic regulatory effects across human tissues. Science.

[64] Satoda M, Zhao F, Diaz GA, Burn J, Goodship J, Davidson HR (2000). Mutations in TFAP2B cause Char syndrome, a familial form of patent ductus arteriosus. Nat Genet.

[65] Coursimault J, Guerrot A-M, Morrow MM, Schramm C, Zamora FM, Shanmugham A (2022). MYT1L-associated neurodevelopmental disorder: description of 40 new cases and literature review of clinical and molecular aspects. Hum Genet.

[66] Schott J-J, Alshinawi C, Kyndt F, Probst V, Hoorntje TM, Hulsbeek M (1999). Cardiac conduction defects associate with mutations in SCN5A. Nat Genet.

[67] Wegerer JV, Helinger B, Berger M, Walden J (1997). A calcium antagonistic effect of the new antiepileptic drug lamotrigine. Eur Neuropsychopharm.

[68] Rogawski MA, Loscher W (2004). The neurobiology of antiepileptic drugs. Nat Rev Neurosci.

[69] Vasconcelos FF, Sessa A, Laranjeira C, Raposo AASF, Teixeira V, Hagey DW (2016). MyT1 counteracts the neural progenitor program to promote vertebrate neurogenesis. Cell Rep.

[70] Wöhr M, Fong WM, Janas JA, Mall M, Thome C, Vangipuram M (2022). Myt1l haploinsufficiency leads to obesity and multifaceted behavioral alterations in mice. Mol Autism.

[71] Chen J, Fuhler N, Noguchi K, Dougherty JD. MYT1L is required for suppressing earlier neuronal development programs in the adult mouse brain. BioRxiv 2022. 10.1101/2022.10.17.512591. 10.1101/gr.277413.122 PMC10234307 37100461

[72] Heyes S, Pratt WS, Rees E, Dahimene S, Ferron L, Owen MJ (2015). Genetic disruption of voltage-gated calcium channels in psychiatric and neurological disorders. Prog Neurobiol.

[73] Andrade A, Brennecke A, Mallat S, Brown J, Gomez-Rivadeneira J, Czepiel N (2019). Genetic associations between voltage-gated calcium channels and psychiatric disorders. Int J Mol Sci.

[74] Avazzadeh S, McDonagh K, Reilly J, Wang Y, Boomkamp SD, McInerney V (2019). Increased Ca2+ signaling in NRXN1α+/− neurons derived from ASD induced pluripotent stem cells. Mol Autism.

[75] Caldwell AB, Liu Q, Schroth GP, Galasko DR, Yuan SH, Wagner SL (2020). Dedifferentiation and neuronal repression define familial Alzheimer’s disease. Sci Adv.

[76] Mertens J, Herdy JR, Traxler L, Schafer ST, Schlachetzki JCM, Böhnke L, et al. Age-dependent instability of mature neuronal fate in induced neurons from Alzheimer’s patients. Cell Stem Cell. 2021;28:1533–48. 10.1016/j.stem.2021.04.004 PMC8423435 33910058

[77] Loo L, Simon JM, Xing L, McCoy ES, Niehaus JK, Guo J (2019). Single-cell transcriptomic analysis of mouse neocortical development. Nat Commun.

