# Supplementary material for: MYT1L haploinsufficiency in human neurons and mice causes autism-associated phenotypes that can be reversed by genetic and pharmacologic intervention
Source: Mol Psychiatry. 2023 Feb 14;28(5):2122–35. doi: 10.1038/s41380-023-01959-7 (PMC10575775; doi:10.1038/s41380-023-01959-7)
Supplement: Supplementary file 1 — Supplementary text [file 41380_2023_1959_MOESM1_ESM.docx]

**FIGURE LEGENDS SUPPLEMENTARY FIGURES**

**Supplementary Fig. 1 Generation and characterisation of *Myt1l* mutant mice.** **(A)** Gene expression analysis of human *MYT1L* and mouse *Myt1l* showed lifelong expression with developmental peak and age-associated decline. Reads per kilobase of transcript per million mapped reads (RPKM) from [1]. **(B)** Exon structure of mouse *Myt1l* on Chromosome 12: 29,578,383-29,973,212 (ENSMUSG00000061911). Exon 6 containing the ATG START codon is highlighted and the DNA sequence is displayed together with the CRISPR/Cas9 guide RNA sequence used to induce mutagenesis. **(C)** Schematic of CRISPR/Cas9-induced 7 bp deletion (dotted red line) in exon 6 inducing a frameshift (red) at amino acid (aa) 11 and terminating in a premature STOP at aa 78 in exon 9 (p.R11GfsX68). Mutant mice were identified by Sanger sequencing, which confirmed a 7-bp deletion in exon 6 of mouse Myt1l. **(D)** PCR with primer pairs P1 (wild type, blue) and P2 (mutant, red) were used to genotype *Myt1l* (+/+), (+/-), and (-/-) mice. **(E)** Normalised read counts of MYT family members *Myt1l, St18* and *Myt1* in MYT1L-deficient mice showed no nonsense-mediated decay of *Myt1l* transcript in RNA-Seq experiments. ** p-adj < 0.01, **** p-adj < 0.0001. **(F)** *Myt1l* transcript tracks confirmed the expected 7-bp deletion in exon 6 of *Myt1l* RNA. **(G)** Western blot analysis showed the expected depletion of full-length MYT1L. A smaller molecular weight band was detected in mutants at E18.5 and P0. **(H)** Representative brain sections of E15.5 mouse cortices from *Myt1l* (+/-) and (-/-) mice displayed expected decrease of nuclear MYT1L immunofluorescence signal compared to controls (+/+). Scale bar 100 μm. **(I)** Immunoprecipitation of endogenous MYT1L from *Myt1l* (*-/-*) and control (+/+) brains followed by mass spectrometry analysis revealed the expected de novo peptides resulting from the frameshift at aa 11 in *Myt1l* (-/-) but not in (+/+) conditions. Unexpectedly we found MYT1L peptide fragments starting after aa 181 in (-/-), suggesting that an internal methionine at aa 99 was used to produce N-terminally truncated (aa 99 - 1187) MYT1L. This isoform would explain the lower molecular weight band in Western blot, but would lack the nuclear localisation signal (NLS) aa 54-82. **(J)** Overexpression of full-length and N-terminally truncated (aa 99 - 1187) *Myt1l* at day *in vitro* 3 (DIV 3) in primary hippocampal cultures shown at DIV 11 by qRT-PCR quantification and representative Western blot analysis. Scatter plot of biological replicates with the median are displayed. **(K)** Nuclear intensity of FLAG-tagged truncated MYT1L (aa 99 – 1187) compared to full-length control at DIV 11 after overexpression as in J. Representative immunofluorescence images with indicated antibodies are shown, scale bar 100 µm. Bar graphs show mean values, data points from individual animals are displayed, error bars = SEM, Mann-Whitney test in panel J, unpaired t-test in panel K ** p < 0.01, **** p < 0.0001.

**Supplementary Fig. 2 Single cell RNA sequencing of MYT1L-deficient prefrontal mouse cortex after birth and CUT&RUN during development. (A)** Single cell RNA-Seq of MYT1L-deficient prefrontal cortices at birth and cell type assignment based on reference data from [2]. **(B)** Normalised transcript levels of *Myt1l* (***+/+***) confirms expression in neuronal cell populations. **(C)** Likelihood analysis of observing MYT1L-deficient (*-/-*, yellow) and control (*+/*+, black) cell enrichment in populations of specific cell types using MELD. **(D)** Beeswarm plot of MELD likelihood analysis for indicated neuronal cell type clusters with individual MYT1L-deficient (*+/-*, teal), (*-/-*, yellow) and control (*+/*+, black) cells. **(E)** Ratio of MYT1L-deficient (*-/-*, yellow) and control (*+/*+, black) cells in indicated cell type clusters. * FDR < 0.01 & abs(Log2FC) > 1. **(F)** Several non-neuronal signatures were increased (red) upon MYT1L depletion in layer II-IV (left) and layer V-IV (right) neurons based on GSEA. Shown are normalised enrichment scores (NES) of signatures with false discovery rate (FDR) ≤ 0.25. FDR * < 0.25, ** < 0.1, *** < 0.01. **(G)** Genome-wide occupancy profiles of endogenous MYT1L in the prefrontal cortex of wildtype mice determined by CUT&RUN at E18.5 (n = 3) and adult stage (n = 3). **(H)** Pie charts indicate the distribution of detected MYT1L-bound sites at annotated genomic regions. **(I)** The MYT1L DNA-binding motif (AAAGTT) is significantly enriched at bound sites at embryonic and adult stage. **(J)** Overlap of the MYT1L target genes (peaks called ± 5 kb from transcription start site) at different time points during development (remaining data for P0 shown in main figure). **(K)** Comparison of genome-wide expression changes and expression changes of significantly deregulated MYT1L target genes (P0 MY1TL CUT&RUN peak ± 5 kb from transcription start site) for indicated single cell populations. Mann-Whitney test **** p < 0.0001. For scRNA-Seq n = 2 for *Myt1l* (*-/-;* 10503 cells), (*+/-*; 13688 cells) and (*+/+*; 12072 cells), respectively.

**Supplementary Fig. 3 Characterisation of brain development and morphology of MYT1L-deficient mice.** **(A)** Quantification of SOX2+ or TBR2+ cells in the cortex of *Myt1l* (*+/-*) and (*-/-*) mice compared to (*+/+*) control at E15.5 across the same area of the entire cortex. Representative images of a ventricular and subventricular zone magnification stained with indicated antibodies are shown. n = 5, scale bar 50 µm. **(B)** Weight of *Myt1l* (+/+), (+/-), and (+/-) pups and brains at P0; n ≥ 6. **(C)** Quantification of absolute cortical length and thickness at two locations in the cortex of *Myt1l* (+/+), (+/-), and (+/-) pups at P0; n ≥ 5 for cortical length; sections of n = 3 for cortical thickness. Red lines indicate location of measured features. Bar graphs show mean values, data points from individual animals or sections are displayed, error bars = SEM, Mann-Whitney t-test * p < 0.05, ** p < 0.01, *** p < 0.001 **** p < 0.0001

**Supplementary Fig. 4 Bulk gene expression changes in *Myt1l-*mutant mice during brain development.** **(A)** Number of deregulated genes upon *Myt1l* mutation across development. Shown are genes that were down- (blue) or upregulated (red) upon MYT1L deficiency with absolute log2 fold change > 1E-05 and p-adj < 0.1. **(B)** Percentage of up- and downregulated genes shown in panel A, which are MYT1L target genes (±5 kb from transcription start site) based on CUT&RUN experiments for the corresponding time points, except all targets pooled for P22. **(C)** Ingenuity Pathway Analysis (IPA) of differentially expressed genes in the cortex of *Myt1l-*mutant mice during development. The state of activity (red) or inhibition (blue) of a signalling pathway or biological function is represented by a z-score (right-tailed Fisher’s exact test). Several pathways and functions were initially repressed and later activated upon MYT1L deficiency. **(D)** GSEA analysis showed depletion of mid-foetal (blue) and enrichment of early-foetal (red) neural development–related gene sets among the genes deregulated in *Myt1l* (*+/-*) and (*-/-*) mutant brains compared to control at E18.5. Several non-neuronal gene signatures were increased (red) in MYT1L-mutant brains at E18.5, P22, and 3 months, while neuronal signatures were decreased (blue) at E18.5 and P22 based on GSEA. Shown are normalised enrichment scores (NES) of signatures with false discovery rate (FDR) ≤ 0.2. FDR * < 0.2, ** < 0.1, *** < 0.01. **(E)** Deregulated genes in MYT1L-deficient mouse brains at E18.5 and P22 display significant (turquoise) overlap with genes linked to epilepsy, schizophrenia, and ASD determined by Fisher’s exact test. **(F)** Overlap of genes up- or downregulated upon *Myt1l* mutation in mouse prefrontal cortex at 3 months and genes that are up- or downregulated at adult stage in other MYT1l and mental disease mouse models [3–5] determined by GeneOverlap. **(G)** Overlap of genes up- or downregulated upon *Myt1l* mutation in mouse cortex at E18.5, P0, P22 and 3 months with genes that are up- or downregulated in ASD-patient brains [6, 7] determined by GeneOverlap. For RNA-Seq analysis n ≥ 5 E18.5, n ≥ 4 P0 for (*+/+*), (*+/-*) and (*-/-*) respectively; n = 6 P22, n ≥ 3 adult for (*+/+*) and (*+/-*) respectively.

**Supplementary Fig. 5 *Myt1l-*mutant mice exhibit behaviour phenotypes.** **(A)** Number of ultrasonic vocalisations (USVs) in *Myt1l (+/-)* mutant compared to control mice at P5 and P8. Pie chart comparing indicated call types at P8 between *Myt1l* mutants and controls; P5: n = 26 for (+/+) and n = 51 for (+/-), P8: n = 25 for (+/+), n = 30 for (+/-). **(B)** Open field experiment analysis of time spent and distance travelled in corners comparing wild type and mutant animals at P23. Distance travelled in centre is displayed separately for male and female mice; n = 25 for (+/+) and n = 29 for (+/-). **(C)** Elevated plus maze observations showed that at P22 *Myt1l*-deficient (*+/-*) mice, due to hyperactivity, spent more time and moved more within open arms and visited both closed and open arms more often compared to control animals; n = 25 for (+/+) and n = 29 for (+/-). **(D)** LABORAS automated home cage observation of indicated features in two months-old mice; n = 24 for (+/+) and n = 29 for (+/-). **(E)** Social chamber experiments showed that female *Myt1l* (*+/-*) mutants, like controls, displayed expected social exploration behaviour for littermates (mates) and novel mice of the same sex at the age of one month; n = 8 for (+/+) and n = 9 for (+/-). **(F)** In marble burying experiments *Myt1l* (*+/-*) mutants exhibited decreased number of burying events, but no significant change in time spent or number of marbles buried compared to controls; n = 25 for (+/+) and n = 28 for (+/-). Behaviour experiments were performed using three independent cohorts. Bar graphs show mean values, data points from individual animals are displayed, error bars = SEM, Mann-Whitney test * p < 0.05, ** p < 0.01, *** p < 0.001, **** p < 0.0001.

**Supplementary Fig. 6 Engineered human embryonic stem cells with a heterozygous conditional knockout allele of *MYT1L.*** **(A)** Exon structure of human *MYT1L* on chromosome 2: 1,789,113-2,331,664 (ENSG00000186487). Exon 6 containing the ATG START codon is highlighted and the DNA sequence is displayed together with the CRISPR/Cas9 guide RNA sequence used to induce mutagenesis. **(B)** Schematic of targeting construct used for homologous recombination in male H1 ES cells with *neomycin* (neo) selection cassette, cre (loxP) and flp (FRT) recombinase-specific recognition sequences, respectively. Flp-induced recombination removed neomycin selection cassette and cre-mediated recombination deleted exon 7, resulting in a frameshift (red) starting in exon 8 at amino acid (aa) 19 and terminating in a premature STOP at aa 69 in exon 9 (p.V19AfsX51). **(C)** Genotyping PCR with primer pairs P1 (left arm) and P2 (right arm) were used to identify successfully targeted clones. Clone C and H, subsequently referred to as clone 1 and clone 2, resulted from two independent targeting experiments and were used for this study. **(D)** Genotyping PCR with primer pairs P3 (loxP/wt), P4 (flp/wt), and P5 (neo) were used to verify successful heterozygous targeting and neomycin cassette removal upon flp-mediated recombination in clone 1 and clone 2. **(E)** Heterozygous targeting was confirmed by Sanger sequencing that showed insertion of loxP sites within one allele and insertion of silent G > A mutation (red box) to remove the PAM site (yellow box) in both alleles of human *MYT1L* clone 1 and clone 2*.* **(F)** Normalised transcript counts of *MYT1L*, *ST18,* and *MYT1L* 1 and 6 weeks after transcription factor-mediated induced human neurogenesis. n = 4 (clone 1) for (*+/fl*) and (+/-), respectively. * p-adj < 0.1, **** p-adj < 0.0001. **(G)** Western blot quantification of MYT1L levels 6 weeks after induction of neurogenesis normalised to control. Representative Western blot images using indicated antibodies are shown; n = 3. Unpaired t-test **** p < 0.0001.

**Supplementary Fig. 7 Gene expression changes upon conditional heterozygous *MYT1L* deletion in human induced neurons.** **(A)** Selected top gene ontology (GO) terms and p-values of genes that are down- (blue) or upregulated (red) upon MYT1L depletion one or six weeks after induced neurogenesis. No significant (ns) GO term enrichment for downregulated (blue) genes at week 6. **(B)** Comparison of top GO terms of down- (blue, left) or upregulated (red, right) genes upon MYT1L depletion in mice (E18.5, P0, P22, adult) and human induced neurons (week 1 and week 2). A detailed list can be found in Table S6. **(C)** Volcano plot for differentially expressed genes in MYT1L-deficient human induced neurons 6 weeks after transcription factor-mediated induced human neurogenesis. Highlighted are genes that are down- (blue) or upregulated (red) upon MYT1L depletion with absolute log2 fold change > 0.2 and p-adj < 0.1. **(D)** The MYT1L DNA binding motif AAAGTT is significantly enriched at up-regulated genes from panel C. **(E)** Clustergram of upregulated genes (rows) upon MYT1L depletion one (left) or six (right) weeks after induced neurogenesis. Red cells in the matrix indicate if a gene is associated with indicated transcription factor (TF) motif, 100 up-regulated genes are displayed based on top 3 enriched TF motifs (columns). **(F)** Several non-neuronal signatures were increased (red) upon MYT1L depletion after transcription factor-mediated human neurogenesis after one and six weeks of differentiation based on GSEA. Shown are normalised enrichment scores (NES) of signatures with false discovery rate (FDR) ≤ 0.2. FDR * < 0.2, ** < 0.1, *** < 0.01. A-E displays data for representative clone 1. **(G)** Ingenuity Pathway Analysis (IPA) of differentially expressed genes in MYT1L-depleted human induced neurons. The state of activation (red) or inhibition (blue) of a pathway or biological function is represented by a z-score (right-tailed Fisher’s exact test). Results are displayed for two clones 1 and 6 weeks after transcription factor-mediated induced human neurogenesis. Clone 1 n = 4 day 7, n = 4 day 43, clone 2 n = 5 day 7, n = 5 day 43 for (*+/fl*) and (*+/-*), respectively.

**Supplementary Fig. 8** **Neuronal** **branch length and bifurcations of human MYT1L-mutant neurons. (A)** Total branch length (sum) and number of bifurcations of *MYT1L* (+/-) human induced neurons 10 days after induced neurogenesis compared to *MYT1L* (+/*fl*) control. **(B)** Total branch length (sum) and number of bifurcations of *MYT1L* (+/-) human induced neurons 6 weeks after induced neurogenesis compared to *MYT1L* (+/*fl*) control. **(C)** Representative traces for (*+/fl,* black) and (+/-, teal) of clone 1 at week 6 after induced neurogenesis are shown. Scale bar 100 µm. Bar graphs show mean values of representative clone 1, number of analysed cells and independent replicates are indicated in the bar graph. Error bars = SEM. Mann-Whitney-test, ns not significant.

**Supplementary Fig. 9 Chemical WNT and NOTCH pathway inhibition can in part rescue transcriptional changes upon MYT1L depletion in induced human neurons.** **(A)** Overlap of genes up- or downregulated upon *MYT1L* mutation in human induced neurons at day 7 with genes that are up- or downregulated in MYT1L-depleted neurons upon WNT and NOTCH inhibition via XAV939 and DAPT based on RNA-Seq and determined by GeneOverlap indicate that deregulated genes in MYT1L-deficient human induced neurons can be partially normalised by inhibitor treatment. n = 8 for (*+/fl*) and (*+/-*), respectively, n = 4 (*+/-*) + each inhibitor treatment, respectively. **(B)** Example genes, which increased or reduced expression in MYT1L-depleted induced human neurons at day 7 that could be restored by inhibitor treatment. n = 4 for (*+/fl*) and all (*+/-*) samples, respectively, DESeq2 analysis * p < 0.05, ** p < 0.01, **** p < 0.0001, ns = not significant. **(C)** Examples of transcription factors that peak in expression early during induced neurogenesis based on published transcriptome data [8] displayed as z-score are initially (week 1) down and later (week 6) significantly upregulated during induced neurogenesis in MYT1L-mutant neurons, indicating delayed neurogenesis. Deregulation of gene expression based on RNA-Seq is displayed as fold change compared to control. n = 4 for (*+/fl*) and (*+/-*), respectively. **(D)** Genes from panel C, which are downregulated in *MYT1L* (+/-) neurons compared to (*+/fl*) control early during development were upregulated upon WNT and NOTCH inhibition via XAV939 and DAPT. Deregulation of gene expression based on RNA-Seq is displayed as fold change compared to control ((*+/fl*) or (*+/-*) without inhibitor), n = 8 for (*+/fl*) and (*+/-*), respectively, n = 4 (*+/-*) + each inhibitor treatment, respectively. All data is from clone 1.

**Supplementary Fig. 10 MYT1L-deficient induced human neurons display network hyperactivity.** **(A)** Inverse correlation of MYT1L protein levels and MEA hyperactivity. MYT1L depletion compared to control was determined by Western blot and plotted against the increase in spikes between depleted and isogenic control neurons for both clone 1 and 2. Effective heterozygous MYT1L depletion (~50%) caused by efficient cre-virus transduction resulted in increased number of spikes compared to suboptimal transduction that resulted in incomplete (< 50%) depletion. **(B)** Increased spike frequency of *MYT1L* (+/-) human induced neurons compared to control on MEA developed early during induced neurogenesis and did not normalise over time. Data from clone 1. **(C)** Compared to control, *MYT1L* (+/-) clone 1 exhibited significant changes in burst and inter-burst intervals. Remaining data for clone 1 shown in the main figure. **(D)** Clone 2, like clone 1, displayed significantly increased spikes and bursts and exhibited a trend towards increased network spikes and decreased inter-burst intervals compared to controls. Bar graphs display mean values with number of MEA wells from indicated biological replicates, error bars = SEM, unpaired t-test for panel B, Mann-Whitney test for panel A, C, D. * p < 0.05, ** p < 0.01, ns = not significant.

**Supplementary Fig. 11 Electrophysiology of primary mouse hippocampal and cortical neurons in culture.** **(A)** Multi-electrode array-based functional analysis of cultured *Myt1l* (*+/-*; teal) and (*-/-*; yellow) mutant neurons derived from the hippocampus of newborn mice over time. Compared to control, *Myt1l* (*+/-*) and (*-/-*) mutant neurons exhibited significantly increased spikes over several days *in vitro* (DIV) along with significant changes in burst and inter-burst intervals. **(B)** Overexpression of N-terminally truncated (aa 99 - 1187) *Myt1l* did not result in electrophysiological hyperactivity on MEA compared to full-length *Myt1l*, indicating no dominant negative effect of the truncated protein. **(C)** Time-resolved MEA analysis of cortex-derived cultured *Myt1l* (*+/-*) and (*-/-*) mutant neurons from newborn mice compared to control. **(D)** Spontaneous excitatory postsynaptic currents (sEPSCs) were recorded at a holding potential of -70 mV for control (+/+) and *Myt1l* (+/-) and (-/-) mutant neurons-derived from the hippocampus of newborn mice at DIV11. Representative sEPSC traces and quantification of mean amplitude and frequency normalised to control. **(E)** Miniature excitatory postsynaptic currents (mEPSCs) of neurons derived as in D were recorded at a holding potential of -70 mV in the presence of tetrodotoxin (TTX) for control *Myt1l* (*+/+*), (*+/-*), and (*-/-*) mutant cells. Representative traces and quantifications are shown. **(F)** Quantification of neuronal branch length and bifurcations in *Myt1l* (*+/-*), and (*-/-*) mutant primary hippocampal cells compared to control at DIV 11. Representative traces are shown, scale bar 100 µm. Bar graphs display mean values with number of wells or cells from indicated biological replicates, error bars = SEM, One way ANOVA for panel A and C-F, Mann-Whitney test for panel B, * p < 0.05, ** p < 0.01, *** p < 0.001, **** p < 0.0001.

**Supplementary Fig. 12 Electrophysiology of CA1 neurons in acute brain slices.** **(A)** Electrophysiological characterisation of CA1 neurons in brain slices derived from 4-6 weeks-old *Myt1l* (+/+) and (+/-) mice showed no difference in measured intrinsic properties of the cells (action potential (AP) firing upon current injection, input resistance (R_in_), half-width, sag ratio and resting membrane potential (RMP)). **(B)** Increased spontaneous inhibitory postsynaptic current (sIPSC) frequency of CA1 pyramidal neurons in acute mouse brain slices. Representative traces, quantification and cumulative distributions are shown, bar graphs display mean values with number of patched cells from indicated biological replicates, error bars = SEM, Mann-Whitney-test * p < 0.05.

**Supplementary Fig. 13 Genetic and pharmacologic intervention can revert phenotypes induced by MYT1L depletion in cultured neurons and mice.** **(A)** Deregulated non-neuronal MYT1L target genes in *MYT1L*-deficient human neurons (week 6) and primary mouse neurons at day *in vitro* 11 (DIV11). Deregulation of gene expression based on RNA-Seq is displayed as fold change compared to control, n = 4 (human) for (*+/fl*) and (*+/-*), n = 3 (mouse) for (+/+) and (+/-), respectively. Tissue expression data from GTEx portal is displayed as z-score. Top 15 deregulated genes are displayed in main figure. **(B)** Representative Western blot images using indicated antibodies show overexpression of MYT1L in primary hippocampal *Myt1l* (+/-) and (-/-) cultures at DIV11 upon transduction at DIV3. **(C)** Upregulation of *Tubb3* and *Myt1l* in primary hippocampal cultures at DIV11 determined by qRT-PCR upon overexpression as in B. Graphs show scatter plots with the median displayed; n ≥ 13. **(D)** *SCN5A* knockdown by shRNAs compared to scrambled control shRNA could be confirmed in primary hippocampal cultures at DIV 11 and human induced neurons 6 weeks after induced neurogenesis by qRT-PCR. **(E)** LABORAS automated home cage observation showed significant increase in rearing and locomotion in mutant mice compared to control littermates. Acute application of 20 mg/kg lamotrigine can normalise this phenotype towards untreated control levels, while lamotrigine treated wildtype mice also display decreased rearing and locomotion; vehicle: n = 19 for (+/+) and n = 25 for (+/-), lamotrigine: n = 17 for (+/+) and n = 21 for (+/-) from three independent cohorts. For qRT-PCR scatter plots with the median are shown. Bar graphs display mean values with number of animals from indicated biological replicates, error bars = SEM, Mann-Whitney test for panel C, unpaired t-test for panel D, Two-way ANOVA for panel E, * p < 0.05, ** p < 0.01, *** p < 0.001, **** p < 0.0001, ns = not significant.

**SUPPLEMENTARY MATERIALS AND METHODS**

**Statistical analysis:** No statistical methods were used to predetermine sample size. Data are presented as means ± SEM unless stated otherwise (all values including statistical analysis are available in Supplementary Table S13). The number of technical and biological replicates are indicated in the figure or in the figure legend and in Supplementary Tables S2-8 for next-generation sequencing analysis. The distribution of the data was determined using Shapiro-Wilk test and based on the results statistical analysis was performed using t-test, Mann-Whitney test or analysis of variance (ANOVA) test with Dunn’s correction for multiple testing. Primary cultures were randomly prepared for the different genotypes. Cells for patch clamp analyses were randomly picked. Mice in behaviour studies were randomly allocated to treatment groups. Other experiments were not randomized. The investigators were blinded to the genotype of the cells for patch clamp recordings. The primary culture preparation was performed blinded to the genotype of the mice. For all other experiments and the analyses, the investigators were not blinded. Illustrations were generated using Affinity Designer and BioRender.

**Targeting and maintenance of human embryonic stem cells:** H1 (male) human embryonic stem cells (ESCs) (WiCell) were maintained feeder-free in mTeSR Plus medium (Stem Cell Technologies). To target *MYT1L,* H1 cells were transfected while seeding onto neomycin and puromycin-resistant SNL feeder cells (Merck) with a plasmid containing CRISPR/Cas9 (pX330-hsMYT1L) and a guide (AGGAGAAGCGGCATCGCACG) against *MYT1L* (ENSG00000186487) exon 6, together with a *MYT1L* targeting construct (pFLIP-hsMYT1L) and a puromycin resistance conferring plasmid (pSico-puro) using FuGENE 6 (Promega) (Supplementary Table S11). The *MYT1L* targeting construct contained *MYT1L* exon 6 and 7 with exon 7 flanked by loxP sites, which resulted in a frameshift and a premature STOP codon upon cre-recombination (Supplementary Fig. S6). Additionally, the targeting construct included a neomycin-resistance gene flanked by frt sites and *MYT1L* homology arms to enhance homologous recombination upon CRISPR/Cas9-mediated mutagenesis. Puromycin (2 µg/ml c.f.) was added 2 days after transfection for 2 days to select transfected cells followed by neomycin (300 µg/ml c.f.) addition for 1 week to select cells with stable expression of the targeting cassette. Resulting ES cell colonies were individually picked and expanded on SNL feeder cells in human ESC medium (DMEM/F12 (L-Glutamine), 20% knockout serum, glutamax, non-essential amino acids, sodium pyruvate and penicillin/streptomycin, bFGF (200 µl 10 µg/ml in 630 ml; all from Invitrogen), beta-mercaptoethanol (Sigma) and 10 µM Y-27632 (Axon Medchem). Correctly targeted colonies were confirmed by PCR screening and Sanger sequencing (Supplementary Fig. S6). To excise the integrated neomycin resistance, 2 clones from independent targeting experiments were selected and transfected while seeding onto puromycin-resistant SNL feeder cells with a plasmid containing flippase (flp-puro) using FuGENE 6 (Promega). Puromycin (2 µg/ml c.f.) was added 2 days after transfection for 2 days to select flippase transfected cells followed by expansion to pick individual subclones on feeder cells in human ES medium containing 10 µM Y-27632. Neomycin-cassette removal in targeted *MYT1L* (*+/fl*) colonies was confirmed by PCR (Supplementary Fig. S6). Cre-recombination resulted in a ~50% reduction in MYT1L protein levels in resulting human neurons (Fig. 4). Cells were tested to be sure they were negative for mycoplasma contamination. Stem cell work was based on the German Stem Cell Act approved by the Robert Koch-Institute.

**Generation of knockout mice:** To generate *Myt1l* mutant mice a guide RNA (AGGAGAAGCGCCAUCGCACAGUUUUAGAGCUAGAAAUAGCAAGUUAAAAUAAGGCUAGUCCGUUAUCAACUUGAAAAAGUGGCACCGAGUCGGUGCUUUU) targeting *Myt1l* (ENSMUSG00000061911) exon 6 was loaded onto Cas9 protein (Synthego) and electroporated into C57BL6/N zygotes following published protocols [9]. Embryos were briefly cultured to confirm viability and then transferred to pseudo-pregnant surrogate mice for gestation. Offspring were screened for frameshift mutations in exon 6 using PCR followed by Sanger sequencing. Founders carrying a 7-bp frameshift mutation in exon 6 were bred with wild type C57BL/6N mice (Jackson Laboratories) to confirm germline transmission. The 7-bp mutation in exon 6 of *Myt1l* is predicted to cause a frameshift at amino acid (aa) 11 that terminates in a premature STOP at aa 79 in exon 9, similar to a reported patient case with a premature STOP at aa 75 [10] (Fig. 1, a and Supplementary Fig. S1). F1 pups from the founder were genotyped by sequencing as above, then bred to generate experimental animals. Subsequent genotyping of each generation was conducted with wildtype and mutant allele specific PCR primers (Supplementary Table S9) using Phusion polymerase and the following PCR conditions: 95°C for 5 min, 95°C for 30 s, 59°C for 30 s, 72°C for 20 s, repeat 2-4 for 35 cycles, 72°C for 5 min, and hold at 4°C. Generation of knockout mice was performed at Stanford University and approved by the Institutional Animal Care and Use Committee. Breeding was approved by the Regierungspräsidium in Karlsruhe (G-193/19).

**MYT1L immunoprecipitation and mass spectrometry:** For each immunoprecipitation, one hemisphere of P0 mice was flash-frozen in liquid nitrogen. The frozen tissue was lysed in 1 mL lysis buffer containing (in mM): 0.5% Tween-20, 50 Tris pH 7.5, 2 EDTA, 1 DTT, 1 PMSF, 5 NaF (all from Sigma), and complete protease inhibitor (Roche) for 15 min at 4 °C and processed for MYT1L immunoprecipitation as described previously[11]. Bound proteins were enzymatically digested and peptides were resolved using the Easy NanoLC1200 fitted with a trapping (Acclaim Pepmap C18, 5μm, 100 Å, 100μm x 2cm) and an analytical column (nanoEase M/Z Peptide BEH C18 Column, 130Å, 1.7 µm, 75 µm X 250 mm) followed by mass spectrometric analysis (Fusion Orbitrap; Thermo Fisher Scientific). The peptides were introduced to the mass spectrometer via a Sharp Singularity Emitter 365 μm OD x 20 μm ID, length of 8 cm (Fossiliontech) and a nano-source spray voltage of 2 kV. The ion transfer tube temperature was set to 275 °C. Full scan MS^1^ spectra were acquired within the range (m/z) of 375-1500 in the Orbitrap detector with a resolution of 120000. The maximum injection time was set to 50 ms and automatic gain control target (AGC) to 1x10^6^ ions. The most abundant ions within a 3 sec cycle time window were selected for fragmentation. Ions with unassigned charges and charges of 1 or >5 were excluded. Dynamic exclusion was set to 40 sec with a mass tolerance of ±10 ppm. For the MS^2^ scans the quadrupole was used with an isolation window of 1.6 m/z. For peptide fragmentation higher-energy collisional dissociation (HCD) was used at 33%. MS^2^ scans were acquired in the linear ion trap that was operated in the rapid ion scan rate with an AGC target of 1x104 ions or a maximum injection of 50 ms. MS^2^ scans were acquired as centroid data type. Data were processed using Maxquant software 2 (version 1.6.17.0). The search was performed against the mouse Uniprot canonical database (downloaded 04/2019) containing only reviewed entries. Enzyme digestion in Maxquant settings was set to Trypsin allowing for maximum of up to 3 missed-cleavages. Protein N-term acetylation, methionine oxidation, deamidation (NQ) were set as variable modifications and carbamidomethylation of cysteine as a fixed modification. Minimum unique peptides option was set to 1. Match between runs was enabled by setting the match time window at 0.4 min. Both intensity-based absolute quantification and label free quantification values were calculated. Peptide and protein hits were filtered at a false discovery rate (FDR) of 1% with a minimal peptide length of 7 amino acids. The reversed sequences of the target database were used as a decoy database. Second peptide search for the identification of the chimeric MS^2^ spectra was enabled. All other Maxquant options were left to their default settings. Maxquant output tables were analysed with Microsoft Excel and R statistical software environment version 4.0.3 (Supplementary Table S1).

**Primary tissue dissection and cell culture:** Mouse glial cells were isolated from forebrains of wild-type CD1 (Jackson Laboratories) at P0 and maintained in MEF media (DMEM; Invitrogen) containing 10% cosmic calf serum (CCS; Hyclone), beta-mercaptoethanol (Sigma), non-essential amino acids, sodium pyruvate and penicillin/streptomycin (all from Invitrogen) and passaged twice before experiments. For primary neuronal cultures, the hippocampus or the cortex of P0 *Myt1l* mutant or control mouse pups were isolated as published previously [12] and cultured on Matrigel (Corning)-coated coverslips or PEI (Polysciences)-laminin (Invitrogen)-coated MEA plates (Axion) in MEM supplemented with 2% B27, 0.04% glucose, 2 mM L-glutamine and 5% FBS (all from Invitrogen) for one day and then in Neurobasal-A medium supplemented with 2% B27, 2 mM L-glutamine and 5% FBS (all from Invitrogen). Three days after plating the medium was supplemented with 2 µM Ara-C (Sigma) and half of the medium was changed every 2 days for the duration of the experiment. All cells were grown at 37 **°**C and 5% CO_2_. For overexpression or knockdown, the cells were transduced with lentivirus encoding indicated constructs on day *in vitro* 3 (DIV3). Doxycycline was added right before transduction on DIV3 to induce transgene expression.

**Virus production:** Lentivirus was produced by transfection of lentiviral backbones containing the indicated transgenes together with third-generation packaging plasmids into HEK293 cells (CRL-1573, ATCC) following the Trono laboratory protocol [13] (Supplementary Table S11). Virus was concentrated from culture supernatant by ultra-centrifugation (23000 rpm, 2 h, 4 **°**C) and either stored at -80° C or used directly for transduction.

**Generation of human neurons:** Human neuron generation by transcription factor overexpression has been described previously [14]. In brief, on day -1 *MYT1L* (+/fl) human ESCs were treated with Accutase (Gibco) and plated on Matrigel (Corning)-coated 6-well plates in mTeSR Plus containing 10 µM Y-27632 (Axon Medchem). While plating, lentivirus encoding doxycycline-inducible Ngn2, rtTA and either cre-recombinase or an inactive truncated Δcre-recombinase, all under control of the ubiquitin promoter, were used to transduce the cells. For shRNA-mediated gene knockdown, the cells were additionally transduced with lentivirus encoding a *SCN5A* targeting or scrambled control shRNA construct. For morphology analysis, cells were additionally transduced with lentivirus encoding labelling with a channelrhodopsin-mCherry construct. On day 0, Doxycycline (2 mg/ml, Sigma) was added to induce Ngn2 expression and retained in the medium until day 14. On day 1, cells were treated with Accutase (Gibco) and plated on Matrigel (Corning)-coated 6-well plates in DMEM/F12 containing N2-supplement, non-essential amino acids (all from Invitrogen), human BDNF (10 ng/ml, PeproTech), human NT-3 (10 ng/ml, PeproTech), and mouse Laminin-1 (0.2 mg/ml, Invitrogen) containing 10 µM Y-27632 (Axon Medchem) and puromycin (2 µg/ml c.f.) was added for 3 days to select infected cells. On day 4, neuronal cells were treated with Accutase and replated in Neurobasal-A medium supplemented with B27/glutamax (Invitrogen) containing BDNF, NT3, Laminin-1, and Ara-C (2 µM, Sigma). For day 7 harvest, neuronal cells were seeded on Matrigel-coated 12-well plates without addition of glia. WNT and NOTCH inhibition was performed by supplementing the media from day 1 to day 4 with either 10 µM N-[N-(3,5-difluorophenacetyl)-l-alanyl]-S-phenylglycine t-butyl ester (DAPT; Sigma), 15 µM Tetrahydro-2-[4-(trifluoromethyl)phenyl]-4H-thiopyrano[4,3-d]pyrimidin (XAV939; Santa Cruz), or both. For functional maturation, neuronal cells were seeded on Matrigel-coated coverslips or PEI-laminin-coated MEA plates (Axion) together with primary mouse glia with media changes every 2 days. After 1 week media was gradually exchanged with MEM supplemented with 0.5% glucose, 0.02% NaHCO3), 2 µM Ara-C (all Sigma), Transferrin (100 µg/ml c.f.; Merck), 5% FBS, B27, 0.5 mM L-glutamine (all Invitrogen), following media changes every 3–4 days for the duration of the experiment. All cells were grown at 37 **°**C and 5% CO_2_.

**EdU labelling and immunostaining of mouse brain sections:** Timed-pregnant females were intraperitoneally injected at E14.5 with EdU (30 mg/kg body weight) (Life Technologies). After 20 h embryonic brains were harvested. Embryonic brains and brains of newborn mice were dissected, fixed in 4% PFA for 24 h and cryoprotected in 30% sucrose in PBS for 48 h. Brains were placed in OCT compound (Fisher HealthCare), frozen using dry ice and sectioned at 20 μm (embryonic) or 50 µm (P0) on a cryostat (Leica Biosystems). EdU detection was performed with the Click-iT EdU Alexa Fluor 488 imaging kit protocol (Life Technologies) according to the manufacturer’s instructions. Immunolabeling was carried out on slide-mounted cryosections following standard protocols and mounted using S3023 Fluorescence Mounting Medium (Agilent). Antigen retrieval of slide-mounted cryosections was performed for SOX2 and TBR2 immunostaining using Antigen Retrieval Citra Plus Solution (BioGenex) according to manufacturer’s protocol. Images were acquired using a Leica TCS SP5 confocal microscope equipped with a 40X / NA 1.3 HCX PL APO oil objective and LAS AF software (Leica) or a Zeiss LSM700 confocal microscope equipped with a 10X / NA 0.3 air objective and using Zen 3.0 Software (black edition; Zeiss). Q-fraction analysis followed previously established practices [4]. In brief, midcortical 200-μm-wide segments were imaged and EdU+Ki67− cells positioned basal to the SVZ counted, followed by the count of all EdU+ cells. The ratio of the two counts represented the Q-fraction. The number of TBR2+ and SOX2+ cells was determined in midcortical 265-µm-wide segments using an in-house developed Fiji macro. All quantifications were carried out at equivalent anteroposterior positions between genotypes.

**Brain morphology analysis:** For analysis of cortical length, P0 mice were decapitated and the brains were removed from the skull and weighed before they were placed in 4% PFA (Bosterbio). After 24 h fixation, the brains were placed next to a ruler and photographed. The maximal cortical anteroposterior length was measured using Adobe Illustrator and normalised to the respective brain weight. For analysis of cortical thickness, P0 mice were decapitated, the brains were removed from the skull and placed in 4% PFA (Bosterbio) for 24 h. After fixation, the brains were cryoprotected in 30% sucrose in PBS (all from Invitrogen) for 3 days and then frozen on dry ice. Frozen brains were cut coronally in 50-µm sections on a cryotome (Leica Biosystems). Sections were stained with indicated antibody and mounted with Vectashield mounting medium (Vectorlabs). Sections were aligned across genotypes using subcortical anatomical landmarks for orientation (hippocampal length, thalamic size), and images at different positions throughout the brain were acquired using the 4× objective of the DMIL LED microscope with the DFC 300G camera and the LAS X software (all from Leica) or the 10× / 0.3 Plan-NEO Ph1 DICIII objective of the Cell Observer with the CCD camera AxioCam and the ZEN 3.0 (blue edition) software (all from Zeiss). Cortical thickness was measured at a 45° angle from the dorsal midline using Adobe Illustrator. For each section the mean of both hemispheres was used.

**Morphology and nuclear localisation analysis in neuronal cultures:** For morphology analysis of human induced neurons, channelrhodopsin-mCherry overexpressing human induced neurons were mixed 1:100 with unlabelled cells while plating on glia. For morphology analysis of mouse primary cultures, cells were transduced at a low multiplicity of infection with lentivirus encoding doxycycline-inducible tdTomato and *rtTA* under control of the Synapsin promoter on DIV3. Doxycycline was added right before transduction on DIV3. Neurons were washed with PBS and fixed using 4% PFA in PBS (Bosterbio) for 10 min at indicated timepoints. Cells were then permeabilised in 0.25% Triton X-100 (Sigma) in PBS for 10 min, blocked in a solution of PBS containing 5% normal goat serum (Jackson ImmunoResearch) for 1 h, followed by incubation with primary antibody diluted in PBS containing 2.5 % normal goat serum overnight at 4 °C. Cells were washed 3 times in PBS containing 2.5% normal goat serum prior to incubation for 1 h at RT with secondary antibodies. Next, cells were washed 3 times in PBS containing 2.5% normal goat serum and incubated for 5 min with 100 ng/ml DAPI (Invitrogen). Finally, cells were washed with deionised water and coverslips were mounted using S3023 Fluorescence Mounting Medium (Agilent). Tile scan imaging of human induced neurons was performed using Zeiss Cell Observer equipped with a 20X / NA 0.8 Plan-Apo DIC II objective and the CCD camera AxioCam. Imaging and stitching of tiles was performed with Zen 3.0 Software (blue edition; Zeiss). Imaging of mouse primary neurons was performed using Axio Scan.Z1 slide scanner (Zeiss) equipped with 20X / NA 0.8 Plan-Apochromat air objective. All settings were kept constant throughout each experiment. Tracing of neurites and morphology analysis were performed with Fiji using SNT plugin [15, 16]. To quantify nuclear FLAG-MYT1L localisation, primary mouse neurons were fixed at DIV 11 upon overexpression of indicated constructs at DIV 3 and stained and imaged as described above. Quantification of nuclear FLAG signal was performed using Fiji. Neuronal nuclei were selected as ROI, the mean FLAG intensity of all neuronal nuclei was measured and background subtraction was performed.

**Behaviour experiments:** The mice were housed in a temperature-controlled vivarium maintained on a 12-h light–dark cycle and tests were conducted during the light cycle. All procedures were performed at the Interdisciplinary Neurobehavioral Core of Heidelberg University and approved by the Regierungspräsidium in Karlsruhe. Distress, as well as the number of animals used, was minimised. No previous analyses were performed on animals used for behavioural testing. We used mixed genotype home cages with 2 - 5 animals per cage and performed the testing on three independent cohorts of *Myt1l* (*+/-*) mice (first cohort: 5 males, 2 females; second cohort: 12 males, 13 females; third cohort: 3 males, 8 females) and (*+/+*) littermates (first cohort: 1 male, 3 females; second cohort: 2 males, 2 females; third cohort: 11 males, 7 females). The rescue experiments were performed on three independent cohorts of *Myt1l* (+/-) mice (first cohort: 6 males, 12 females; second cohort: 10 males, 18 females; third cohort: 1 male, 5 females) and (+/+) littermates (first cohort: 9 males, 7 females; second cohort: 16 males, 4 females; third cohort: 3 male, 3 females). The mice were injected intraperitoneally with the drug (20 mg/kg lamotrigine) or vehicle (5% PEG 400 in NaCl) 60 min before each behaviour test. At P4, animals were marked by foot tattoo with non-toxic animal tattoo ink (Ketchum Mfg). The SHIRPA procedure was performed at P23 on all animals to exclude motoric or sensory defects of mutant mice.

Ultrasonic vocalisation (USV): P5 and P8 pups were isolated from the mother and placed in an empty glass container (6 cm × 9.5 cm × 7.5 cm). USV was recorded for 5 min using an UltraSoundGate condenser microphone (CM16/CMPA, Avisoft Bioacoustics) placed 30 cm above the testing arena. The microphones were connected to a computer via an Avisoft UltraSoundGate USG416H audio device. The MATLAB package DeepSqueak was used for counting and categorization of the calls [17].

Elevated plus maze (EPM): The EPM is a cross-shaped platform (grey opaque plastic material) with equally sized arms (6 cm × 35 cm) and a central intersection (6 cm × 6 cm), allowing animals to move freely into each zone of the maze. Two of the arms (opposing each other) are flanked by 17 cm opaque walls; the remaining two arms are without walls. The EPM is elevated 70 cm above the floor. At P22, animals were placed in the central intersection and allowed to explore the maze with an illumination of about 80 lux for 10 min. Movements were recorded with a top-mounted video camera and analysed using the tracking software SYGNIS TRACKER.

Open field (OF): The OF is a test for motor activity and anxiety-like behaviour in mice and was performed on P23. In this test, mice were placed in the middle of a white wooden box (60 cm × 60 cm × 30 cm) with an illumination of about 80 lux for 10 min. Movements were recorded with a top-mounted video camera and analysed using the tracking software SYGNIS TRACKER. For the drug treatment, the mice were injected intraperitoneal (i.p.) with 20 mg/kg lamotrigine (Sigma-Aldrich) or vehicle (polyethylenglycol, VWR international) in NaCl 1 h before measurement.

Social chamber test (SCT): SCT was designed to assess social interaction of the test mouse with a familiar littermate and an unfamiliar mouse and was performed with one-month-old mice. The apparatus is composed of a transparent plexiglass box (20 cm × 61 cm × 40 cm) with three compartments including openings between the compartments. A wire mesh cylinder was placed in the left and right compartment. The test was subdivided into habituation time and two trials, 5 minutes each. In the habituation time, the testing mouse was introduced into the apparatus with free access to all compartments. In trial 1 (social recognition), a littermate mouse of the same sex (isolated 12 h before the trial) was placed into the right wire mesh cylinder and the subject mouse was allowed to explore all compartments. In trial 2 (social discrimination), an unfamiliar mouse of the same sex from a different strain (NMRI) was placed into the left mesh wire cylinder and the subject mouse was again allowed to freely explore all compartments. Movements were recorded with a top-mounted video camera and analysed using the tracking software SYGNIS TRACKER.

Marble Burying: Two-months-old animals were habituated in a standard cage (29 cm × 22 cm × 14 cm) for 5 min. Afterwards, 9 standard glass toy marbles (assorted styles and colours, 15 mm diameter, 5.2 g in weight) were put on the surface of the bedding in 3 rows of 3 marbles. The digging behaviour of the animal was monitored for 5 min with a video camera. Individual burying events, time spent burying and the number of buried marbles was manually analysed.

LABORAS: The LABORAS home cage observation (Metris, Netherlands) is a system that uses a carbon fibre platform to detect behaviour-specific vibration patterns produced by the animal. A home cage is placed on top of the platform, and the specific software processes the produced vibrations into various behavioural parameters. We monitored two-months-old animals over 5 h. Animals were placed individually in the calibrated cage under standard housing condition with free access to food and water. For the drug treatment, the mice were injected i.p. with 20 mg/kg lamotrigine (Sigma-Aldrich) or vehicle (polyethylenglycol, VWR international) in NaCl before being placed in the cage.

**Transcriptome analysis using RNA-sequencing:** To collect primary mouse brain samples, the cortex of E18.5 or the prefrontal cortex of P0, P22, and 3 months-old mice was dissected and placed into TRIzol (Invitrogen). Human neurons were harvested from pure cultures at day 7 or upon co-culture with primary mouse glia at day 43 by addition of TRIzol to the cultures (Invitrogen). RNA harvested in TRIzol (Invitrogen) was isolated using the RNA Miniprep kit (Zymo Research). For RNA-sequencing, libraries were prepared following the dUTP protocol [18] and paired-end sequencing reads (100 bp) were generated on NovaSeq 6000 platforms (Illumina). Raw reads were mapped to the reference genomes hg38 or mm10 using STAR [19] and differential expression determined using DESeq2 [20] (R package version 1.28.1) with size factor normalisation and Wald significance tests. For bulk mouse data, we used genetic sex as a covariate. For the human iN samples, we used sample ID as a covariate and computationally removed mouse reads for the 6-weeks samples. Raw p-value histograms were used to verify that the assumptions of the Wald test were satisfied (here: N(0, 1) null distribution for gene dispersions) before continuing. If this was not the case, we estimated the variance of the null–model from the test statistics using the package fdrtool [21] (version 1.2.15) using the Wald statistics as input (empirical null modelling). The p-values were then recalibrated based on the empirically-estimated null variance. EnhancedVolcano (version 1.8.0) (https://github.com/kevinblighe/EnhancedVolcano) was used to make volcano plots, and pheatmap (version 1.0.12) (http://CRAN.R-project.org/package=pheatmap) was used to generate heatmaps. Tissue gene expression data were obtained from the Genotype-Tissue Expression (GTEx) portal (https://gtexportal.org) [22]. GSEA was performed using signatures for specific cell types derived either from tabula muris using the top 500 most highly-expressed unique genes within each cell type, or Lander and PanglaoDB-derived signatures including the top 500 genes from each list [23–26] (Supplementary Table S12). Significance for GSEA profiles were determined by an FDR < 0.25 as described [27]. Pathway enrichment analysis was performed using Ingenuity Pathway Analysis (IPA) [28] and significantly enriched pathways were identified with the criterion p-value < 0.1 using Fisher’s exact test. For functional enrichment analysis significantly deregulated genes (p-adj < 0.1) were analysed using g:Profiler [29]. Odds ratio and gene overlap analysis was performed using the GeneOverlap R package (version 1.26.0) [30]. Overlap of deregulated genes with disease risk genes was manually determined and tested for significance using the Fisher’s exact test in R. Motif enrichment analysis at upregulated genes was performed using Enrichr and volcano plots were generated using Appyter [31, 32].

For single cell RNA-Seq experiments, prefrontal cortices from P0 mice were extracted and dissociated into single cells following the protocol for primary cultures, washed in HBSS + 0.04% BSA (Invitrogen), fixed in ice-cold 80% methanol and stored at -20° C. Barcoding oligonucleotides were designed following the ECCITE-Seq [33] scheme with an amine group at the 5’ end (Supplementary Table S9), which was used to chemically label the fixed cells following a previously published protocol [34]. We pooled ~20.000 cells from two *Myt1l* (*+/+*), (*+/-*), and (*-/-*) animals, respectively. Each pool was loaded into an individual lane of the Chromium Controller (10x Genomics). The Chromium Single Cell 5' v1 reagent kit was employed to process the samples according to the manufacturer’s instructions, with modifications in the cDNA and library preparation steps suggested by the ECCITE-Seq protocol to generate the barcoded oligonucleotide libraries in parallel. Expression and barcode libraries were then diluted to equimolar amounts, pooled in a 9 to 1 ratio and sequenced using a NovaSeq 6000 (Illumina). Single cell RNA-Seq data was analysed using 10x Genomics Cell Ranger (version 4.0.0) [35], Seurat (version 4.0) [36], and Scanpy (version 1.6.0) [37]. Cells containing fewer than 200 or greater than 4000 features, or with mitochondrial genes making up over 15% of genes, were discarded. We used scArches (v0.3.5) [38] with the scANVI model [39] to calculate latent space of dimension ten, and to assign cell type annotations by mapping our data onto reference data [2] using default parameters. Assignments were verified by marker expression. Leiden clustering was performed on latent space (default parameters, k = 15). Two clusters with average annotation probability scores <80% were annotated based on marker gene expression; microglia with *Tmem119* and *Aif1*, and blood cells with *Hba-a1* and *Hbb-bs*. These were removed for downstream analysis. Sub-populations of each cell type most affected by *Myt1l* mutation were identified using MELD (version 1.0) [40] with parameters Beta=31 and KNN=5, which were determined using grid search. To calculate the cell type ratio in our *Myt1l* mutant we normalised the number of cells assigned to each cell type by the total number of cells detected in each condition. We then determined the cell type ratios between conditions based on these normalized values. Significant changes in cell type ratios were identified using a bootstrapping method [41]. Significant differences were defined with an FDR < 0.01 and abs(Log2FC) > 1. Differential expression was performed on these sub-populations using MAST (version 1.16.0) [42] (Supplementary Table S2) within Seurat’s FindMarkers function (logfc.threshold = 0, min.pct = 0.05, all other parameters default). GSEA was performed as described above. Sequencing reads are available on NCBI GEO GSE171327.

**Chromatin binding using CUT&RUN:** The prefrontal cortex of E18.5, P0 and 3-months-old mice was prepped and single cells were prepared following the protocol for primary culture preparation. 300,000 cells per animal were washed twice with wash buffer (20 mM HEPES-KOH pH 7.5, 150 mM NaCl, 0.5 mM spermidine, and 1X Roche Complete Protease Inhibitor), then resuspended in wash buffer and added to concanavalin-A beads (Polysciences) pre-activated with cold binding buffer (20 mM HEPES-KOH pH 7.5, 10 mM KCl, 1 mM CaCl_2_, and 1 mM MnCl_2_). The bead-cell suspension was rotated at RT for 10 minutes and then split into two vials. Supernatant was removed on magnet and the beads resuspended in antibody buffer (0.2 mM EDTA, 0.05% w/v digitonin in wash buffer). Primary antibody (rabbit anti-MYT1L) or control (rabbit IgG) was added at 1:100 v/v final concentration, and the cells were incubated on a nutator at 4 °C for 2 h. Beads were washed twice with digitonin-wash buffer (0.05% w/v digitonin in wash buffer), resuspended in 700 ng/mL pAG-MNase (Protein Expression and Purification Core Facility, EMBL, Heidelberg) in digitonin-wash buffer, and rotated at 4 °C for 1 hour. Beads were washed twice with digitonin-wash buffer followed by resuspension in digitonin-wash buffer and then placed on ice. 1 µL of 100 mM CaCl_2_ was added to initiate chromatin digestion, and the mixture was incubated on ice for 30 min. 50 µL of 2x stop buffer (340 mM NaCl, 20 mM EDTA, 4 mM EGTA, 0.05% w/v digitonin, 50 µg/mL RNase A, 50 µg/mL glycogen, 0.5 ng/mL spike-in E. coli DNA) was added, and the bead suspension incubated at 37 °C for 10 min to release fragments from cells. The supernatant was subjected to phenol-chloroform extraction, and purified DNA fragments were used for library preparation and sequencing. For sequencing, libraries were prepared based on library preparation manual of NEBNext DNA Library Prep Kit for Illumina (NEB E7645), with specific modifications to make libraries from small DNA fragments (dx.doi.org/10.17504/protocols.io.wvgfe3w) and paired-end sequencing reads (40 bp) were generated on NextSeq 2000 platforms (Illumina). CUT&RUN data was analysed using the nf-core/cutandrun pipeline v1.0 (10.5281/zenodo.5653535). The Venn diagram tool from Bioinformatics & Evolutionary Genomics in Gent was used (http://bioinformatics.psb.ugent.be/webtools/Venn/). Homer findMotifsGenome.pl was run on the peak files with the parameters -size -75,75 -mask -mknown and the MYT1L motif AAAGTTW (http://homer.ucsd.edu/homer/).

**Brain slice preparation and electrophysiology:** The mice (4 – 6 weeks postnatal) were deeply anesthetized with the volatile anaesthetic isoflurane and transcardially perfused with ~30 ml NMDG aCSF containing the following (in mM) 92 NMDG, 2.5 KCl, 1.25 NaH_2_PO_4_, 30 NaHCO_3_, 20 HEPES, 25 glucose, 2 thiourea, 5 Na-ascorbate, 3 Na-pyruvate, 0.5 CaCl_2_ and 10 MgCl_2_, oxygenated with carbogen gas (95% O_2_ and 5% CO_2_, pH 7.3-7.4). Mice were decapitated and brains were removed. Transverse hippocampal slices of 400 µm thickness from both hemispheres were cut on a slicer (HR2; Sigmann Elektronik, Germany) in oxygenated NMDG aCSF. Slices were initially recovered in oxygenated NMDG aCSF at 34±1 °C for 11 min followed by incubation in HEPES holding aCSF containing the following (in mM) 92 NaCl, 2.5 KCl, 1.25 NaH_2_PO_4_, 30 NaHCO_3_, 20 HEPES, 25 glucose, 2 thiourea, 5 Na-ascorbate, 3 Na-pyruvate, 2 CaCl_2_ and 2 MgCl_2_, oxygenated at 23±1 °C until used. During experiments, slices were placed in a recording chamber and superfused with oxygenated aCSF containing (in mM): 125 NaCl, 25 NaHCO_3_, 1.25 NaH_2_PO_4_, 2.5 KCl, 25 glucose, 2 CaCl_2_ and 1 MgCl_2_ at 31±1 °C. CA1 pyramidal cells were identified by large somata in the stratum pyramidale with accommodating firing pattern under infrared and differential interference contrast microscope (Olympus BX51WI) [43]. The patch electrodes (3–6 MΩ) were pulled from borosilicate glasses with a filament (O.D. 1.5 mm, I.D. 0.86 mm; Science Products, Germany). For spontaneous EPSCs measurements, pyramidal cells were voltage-clamped at -70 mV with a K^+^-based internal solution containing the following (in mM): 130 K-gluconate, 10 Na-gluconate, 10 HEPES, 10 phosphocreatine, 4 NaCl, 4 MgATP, 0.3 GTP and 0.5% biocytin. For spontaneous IPSCs measurements, putative pyramidal cells were voltage-clamped at 0 mV with a Cs^+^-based internal solution containing the following (in mM): 126 Cs-gluconate, 4 Cs-Cl, 10 HEPES, 10 phosphocreatine, 4 MgATP, 0.3 GTP and 2.5 QX-314 in the presence of CNQX (10 μM) and D-APV (50 μM). All slice recordings were performed using an EPC 10 amplifier (HEKA, Germany) and data were acquired by using Patchmaster software (HEKA, Germany). Pipette capacitance was maximally compensated. Series resistance (≤ 30 MΩ) was partially compensated in voltage-clamp mode (correction 70-80% with a 10-µs lag) and fully compensated in current-clamp mode (correction 100% with a 10-µs lag). Data were excluded if the series resistance changed more than 20%. Liquid junction potentials were not corrected. Signals were filtered at 3 kHz and sampled at 20 kHz. Recordings of excitatory synaptic currents in primary hippocampal cultures at DIV11 were performed in voltage-clamp mode at -70 mV holding potential, with an internal solution containing (in mM): 130 Cs-Gluconate, 5 TEA-Chloride, 0.2 EGTA, 4 Mg-ATP, 0.3 Na-GTP, 12 Na-Phosphocreatin, and 10 HEPES-NaOH (pH adjusted to 7.4, 315 mOsm) in the presence or absence of tetrodotoxin (TTX). The following extracellular solution was used (in mM): 125 NaCl, 2.5 KCl, 25 NaHCO_3_, 0.4 Ascorbic acid, 3 Myo-inositol, 2 Sodium Pyruvate, 1.25 NaH_2_PO_4_.2H_2_0, 2 CaCl_2_, 1 MgCl_2_, 25 D(+)-glucose (pH 7.4, 315 mOsm). Culture recordings were performed using a Multiclamp 700B amplifier (Molecular Devices) controlled with Clampex 10.4 or a dual-channel HEKA amplifier controlled by Patchmaster 10 software. Culture data were analysed offline using Clampfit 10.4 and custom-written macros in IgorPro (Wavemetric). Slice EPSCs were firstly automatically detected by Mini Analysis (Synaptosoft) and then inspected manually. All recordings were performed blinded with cultures and animals originating of the same litter analysed in parallel.

**Multi-electrode array (MEA) and drug treatment:** MEA measurements were performed using the Maestro Pro multiwell device with the Axis Navigator software and 48-well plates containing 16 electrodes per well (all from Axion Biosystems). The data was acquired using a sampling rate of 12.5 kHz and filtered using a 200−3000 Hz Butterworth bandpass filter. The detection threshold was set to 6 × standard deviation of the baseline electrode noise. After equilibration for 20 min, spontaneous activity was recorded for 10 min at the indicated days. For acute treatments of human induced or mouse hippocampal neurons, 10 µM lamotrigine (Targetmol) was added per well and the plate was measured before and 2 h after the treatment. The spike raster plots were produced using the Neural Metric Tool (Axion Biosystems). The spike list files were used to analyse the data in R Studio with the MEA analysis package meaRtools for interpretation of neuronal activity patterns [44]. Wells in which fewer than 4 electrodes were active (> 1 spike / min) were considered inactive and removed from analyses. Bursts were detected with the maximum interval algorithm (min number of spikes: 5, max inter-spike interval: 100 ms). If neurons on ≥ 3 electrodes fired in a time window of ≤10 ms, it was considered a network spike.

**Quantitative real time polymerase chain reaction (qRT-PCR):** Samples were harvested in TRIzol (Thermo Fisher Scientific) and RNA was isolated using the Quick RNA Miniprep kit (Zymo Research) following the manufacturer’s instructions. Extracted RNA samples were stored at -80°C until use. cDNA synthesis was performed with the LunaScript RT Super Mix Kit (NEB) according to the manufacturer’s guidelines. For each qPCR reaction, 5 μL Power SYBR Green PCR Master Mix (Thermo Fisher Scientific) were mixed with 2.1 μL nuclease-free water, 0.4 μL 10 μM primer mix, containing 5 μM forward and 5 μM reverse primer (Table S9) and 2.5 μL cDNA. Quantitative assessment of gene expression was performed in a 384-well format with the QuantStudio5 Real Time PCR system (Thermo Fisher Scientific).

**SDS Polyacrylamide Gel Electrophoresis (SDS-PAGE) and Western blot:** Cultured cells were directly harvested in SDS-PAGE sample buffer containing benzonase (1:100, Merck). Brain samples were lysed in RIPA buffer containing complete protease inhibitor (1:100, Roche), sonicated and concentrations were determined using a BSA standard (Merck) and a BCA protein assay (Thermo Fisher Scientific). Samples were separated using NuPAGE 4-12% Bis-Tris gels (Thermo Fisher Scientific) in MOPS SDS running buffer (Thermo Fisher Scientific). Proteins were transferred onto nitrocellulose membranes in transfer buffer (Thermo Fisher Scientific) using a wet transfer system. For normalisation, total protein stain was performed with Revert-700 total protein stain according to manufacturer’s protocol (LICOR). Proteins were detected with corresponding antibodies (Table S10) using the ODYSSEY CLx Imaging System (LI-COR).

**Plasmid constructs:** DNA constructs were generated by DNA synthesis (IDT) followed by ligation into restriction digested vectors using indicated enzymes and T4 DNA ligase (all from NEB). A complete list of all constructs and primers generated in this study can be found in Supplementary Table S9&11.

**Antibodies:** A complete list of all primary antibodies used in this study can be found in Supplementary Table S10. Secondary Alexa-conjugated antibodies (all from Invitrogen) were used at 1:2000 for immunostaining of cultured cells and 1:400 for brain sections and secondary IRDye-conjugated antibodies were used at 1:10000 (all from LI-COR).

REFERENCES

1. Cardoso-Moreira M, Halbert J, Valloton D, Velten B, Chen chunyan, Shao Y, et al. Gene expression across mammalian organ development. Nature. 2019;571:505–509.

2. Loo L, Simon JM, Xing L, McCoy ES, Niehaus JK, Guo J, et al. Single-cell transcriptomic analysis of mouse neocortical development. Nat Commun. 2019;10:1--11.

3. Chen J, Lambo ME, Ge X, Dearborn JT, Liu Y, McCullough KB, et al. A MYT1L syndrome mouse model recapitulates patient phenotypes and reveals altered brain development due to disrupted neuronal maturation. Neuron. 2021;109:3775-3792.e14.

4. Gompers AL, Su-Feher L, Ellegood J, Copping NA, Riyadh MA, Stradleigh TW, et al. Germline Chd8 haploinsufficiency alters brain development in mouse. Nat Neurosci. 2017;20:1062--1073.

5. Kim JG, Armstrong RC, Agoston D v, Robinsky A, Wiese C, Nagle J, et al. Myelin transcription factor 1 (Myt1) of the oligodendrocyte lineage, along with a closely related CCHC zinc finger, is expressed in developing neurons in the mammalian central nervous system. J Neurosci Res. 1997;50:272–290.

6. Voineagu I, Wang X, Johnston P, Lowe JK, Tian Y, Horvath S, et al. Transcriptomic analysis of autistic brain reveals convergent molecular pathology. Nature. 2011;474:380--386.

7. Katayama Y, Nishiyama M, Shoji H, Ohkawa Y, Kawamura A, Sato T, et al. CHD8 haploinsufficiency results in autistic-like phenotypes in mice. Nature. 2016;537:675--679.

8. Lin H-C, He Z, Ebert S, Schörnig M, Santel M, Nikolova MT, et al. NGN2 induces diverse neuron types from human pluripotency. Stem Cell Rep. 2021;16:2118–2127.

9. Chen S, Lee B, Lee AYF, Modzelewski AJ, He L. Highly efficient mouse genome editing by CRISPR ribonucleoprotein electroporation of zygotes. J Biol Chem. 2016;291:14457--14467.

10. Windheuser IC, Becker J, Cremer K, Hundertmark H, Yates LM, Mangold E, et al. Nine newly identified individuals refine the phenotype associated with MYT1L mutations. Am J Med Genet A. 2020;182:1021--1031.

11. Mall M, Kareta MS, Chanda S, Ahlenius H, Perotti N, Zhou B, et al. Myt1l safeguards neuronal identity by actively repressing many non-neuronal fates. Nature. 2015;544:245–249.

12. Maximov A, Pang ZP, Tervo DGR, Sudhof TC. Monitoring synaptic transmission in primary neuronal cultures using local extracellular stimulation. J Neurosci Meth. 2007;161:75--87.

13. Dull T, Zufferey R, Kelly M, Mandel RJ, Nguyen M, Trono D, et al. A Third-Generation Lentivirus Vector with a Conditional Packaging System. J Virol. 1998;72:8463--8471.

14. Zhang Y, Pak CH, Han Y, Ahlenius H, Zhang Z, Chanda S, et al. Rapid single-step induction of functional neurons from human pluripotent stem cells. Neuron. 2013;78:785--798.

15. Schindelin J, Arganda-Carreras I, Frise E, Kaynig V, Longair M, Pietzsch T, et al. Fiji: an open-source platform for biological-image analysis. Nat Methods. 2012;9:676–682.

16. Arshadi C, Günther U, Eddison M, Harrington KIS, Ferreira TA. SNT: a unifying toolbox for quantification of neuronal anatomy. Nat Methods. 2021;18:374–377.

17. Coffey KR, Marx RG, Neumaier JF. DeepSqueak: a deep learning-based system for detection and analysis of ultrasonic vocalizations. Neuropsychopharmacol. 2019;44:859--868.

18. Levin JZ, Yassour M, Adiconis X, Nusbaum C, Thompson DA, Friedman N, et al. Comprehensive comparative analysis of strand-specific RNA sequencing methods. Nat Methods. 2010;7:709--715.

19. Dobin A, Davis CA, Schlesinger F, Drenkow J, Zaleski C, Jha S, et al. STAR: ultrafast universal RNA-seq aligner. Bioinformatics. 2013;29:15--21.

20. Love MI, Huber W, Anders S. Moderated estimation of fold change and dispersion for RNA-seq data with DESeq2. Genome Biol. 2014;15:550.

21. Strimmer K. A unified approach to false discovery rate estimation. Bmc Bioinformatics. 2008;9:303.

22. Consortium TGte. The GTEx Consortium atlas of genetic regulatory effects across human tissues. Science. 2020;369:1318–1330.

23. Franzen O, Gan LM, Bjorkegren JLM. PanglaoDB: A web server for exploration of mouse and human single-cell RNA sequencing data. Database. 2019;2019.

24. Schaum N, Karkanias J, Neff NF, May AP, Quake SR, Wyss-Coray T, et al. Single-cell transcriptomics of 20 mouse organs creates a Tabula Muris. Nature. 2018;562:367--372.

25. Schiebinger G, Shu J, Tabaka M, Cleary B, Subramanian V, Solomon A, et al. Optimal-Transport Analysis of Single-Cell Gene Expression Identifies Developmental Trajectories in Reprogramming. Cell. 2019;176:928--943.e22.

26. Trapnell C, Williams BA, Pertea G, Mortazavi A, Kwan G, Baren MJV, et al. Transcript assembly and quantification by RNA-Seq reveals unannotated transcripts and isoform switching during cell differentiation. Nat Biotechnol. 2010;28:511--515.

27. Subramanian A, Tamayo P, Mootha VK, Mukherjee S, Ebert BL, Gillette MA, et al. Gene set enrichment analysis: A knowledge-based approach for interpreting genome-wide expression profiles. P Natl Acad Sci Usa. 2005;102:15545--15550.

28. Kramer A, Green J, Pollard J, Tugendreich S. Causal analysis approaches in Ingenuity Pathway Analysis. Bioinformatics. 2014;30:523--530.

29. Raudvere U, Kolberg L, Kuzmin I, Arak T, Adler P, Peterson H, et al. G:Profiler: A web server for functional enrichment analysis and conversions of gene lists (2019 update). Nucleic Acids Res. 2019;47:W191--W198.

30. Shen L. GeneOverlap: An R package to test and visualize gene overlaps. 2014.

31. Kuleshov MV, Jones MR, Rouillard AD, Fernandez NF, Duan Q, Wang Z, et al. Enrichr: a comprehensive gene set enrichment analysis web server 2016 update. Nucleic Acids Res. 2016;44:W90--W97.

32. Chen EY, Tan CM, Kou Y, Duan Q, Wang Z, Meirelles GV, et al. Enrichr: Interactive and collaborative HTML5 gene list enrichment analysis tool. Bmc Bioinformatics. 2013;14:128.

33. Mimitou EP, Cheng A, Montalbano A, Hao S, Stoeckius M, Legut M, et al. Multiplexed detection of proteins, transcriptomes, clonotypes and CRISPR perturbations in single cells. Nat Methods. 2019;16:409--412.

34. Gehring J, Park JH, Chen S, Thomson M, Pachter L. Highly multiplexed single-cell RNA-seq by DNA oligonucleotide tagging of cellular proteins. Nat Biotechnol. 2020;38:35--38.

35. Zheng GXY, Terry JM, Belgrader P, Ryvkin P, Bent ZW, Wilson R, et al. Massively parallel digital transcriptional profiling of single cells. Nat Commun. 2017;8:1--12.

36. Hao Y, Hao S, Andersen-Nissen E, Mauck WM, Zheng S, Butler A, et al. Integrated analysis of multimodal single-cell data. Biorxiv. 2020:2020.10.12.335331.

37. Wolf FA, Angerer P, Theis FJ. SCANPY: Large-scale single-cell gene expression data analysis. Genome Biol. 2018;19:15.

38. Lotfollahi M, Naghipourfar M, Luecken MD, Khajavi M, Buttner M, Avsec Z, et al. Query to reference single-cell integration with transfer learning. Biorxiv. 2020:2020.07.16.205997.

39. Xu C, Lopez R, Mehlman E, Regier J, Jordan MI, Yosef N. Probabilistic harmonization and annotation of single‐cell transcriptomics data with deep generative models. Mol Syst Biol. 2021;17:e9620.

40. Burkhardt DB, Stanley JS, Tong A, Perdigoto AL, Gigante SA, Herold KC, et al. Quantifying the effect of experimental perturbations at single-cell resolution. Nat Biotechnol. 2021:1--11.

41. Miller SA, Policastro RA, Sriramkumar S, Lai T, Huntington TD, Ladaika CA, et al. LSD1 and Aberrant DNA Methylation Mediate Persistence of Enteroendocrine Progenitors That Support BRAF-Mutant Colorectal Cancer. Cancer Res. 2021;81:3791–3805.

42. Finak G, McDavid A, Yajima M, Deng J, Gersuk V, Shalek AK, et al. MAST: A flexible statistical framework for assessing transcriptional changes and characterizing heterogeneity in single-cell RNA sequencing data. Genome Biol. 2015;16:278.

43. Liu Y-C, Cheng J-K, Lien C-C. Rapid Dynamic Changes of Dendritic Inhibition in the Dentate Gyrus by Presynaptic Activity Patterns. J Neurosci. 2014;34:1344–1357.

44. Gelfman S, Wang Q, Lu Y-F, Hall D, Bostick CD, Dhindsa R, et al. meaRtools: An R package for the analysis of neuronal networks recorded on microelectrode arrays. Plos Comput Biol. 2018;14:e1006506.
