# Supplementary figures and images for: MYT1L haploinsufficiency in human neurons and mice causes autism-associated phenotypes that can be reversed by genetic and pharmacologic intervention
Source: Mol Psychiatry. 2023 Feb 14;28(5):2122–35. doi: 10.1038/s41380-023-01959-7 (PMC10575775; doi:10.1038/s41380-023-01959-7)

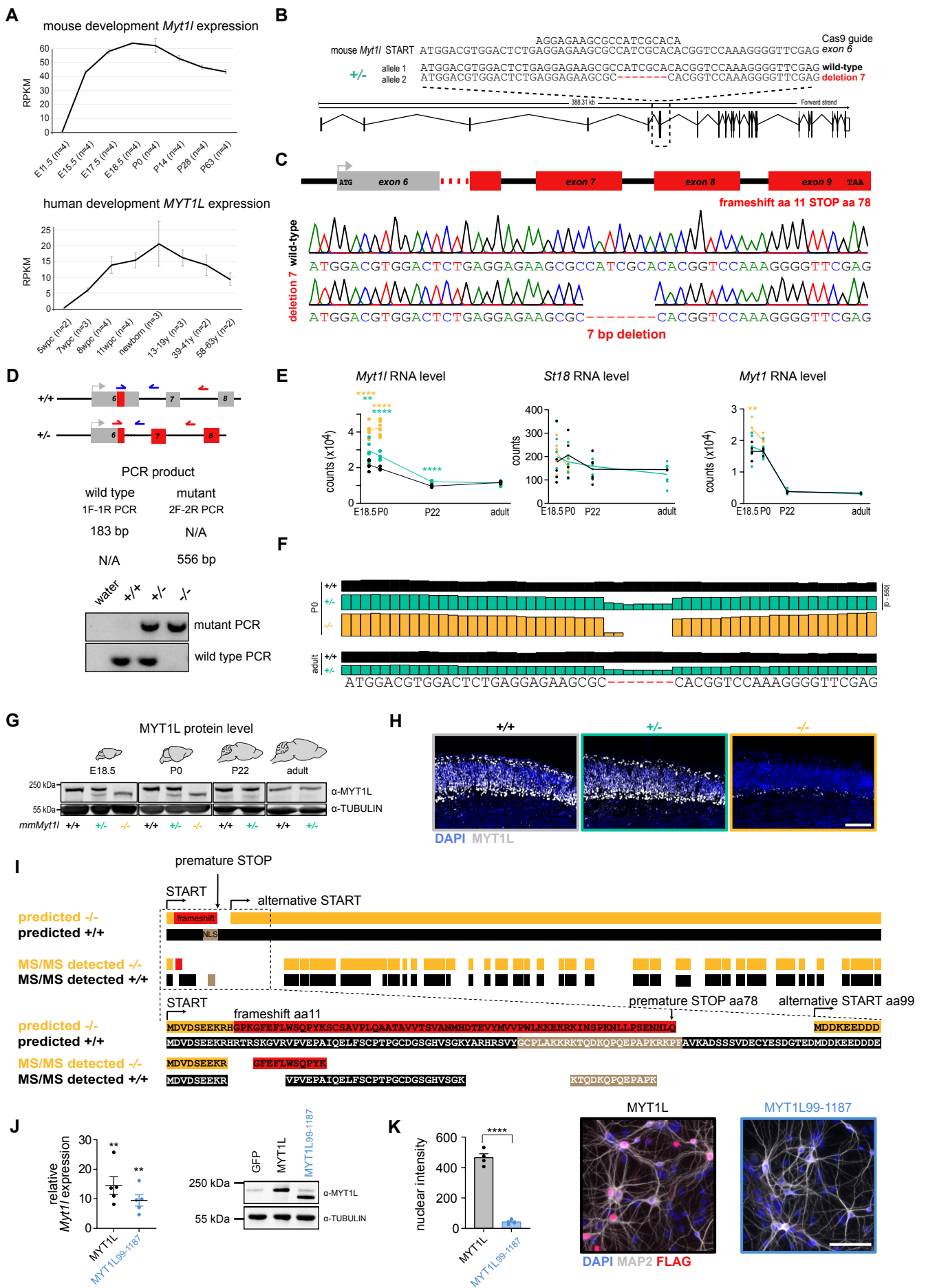

Supplement: Supplementary file 2 — Supplementary Figure 1 [file 41380_2023_1959_MOESM2_ESM.pdf]

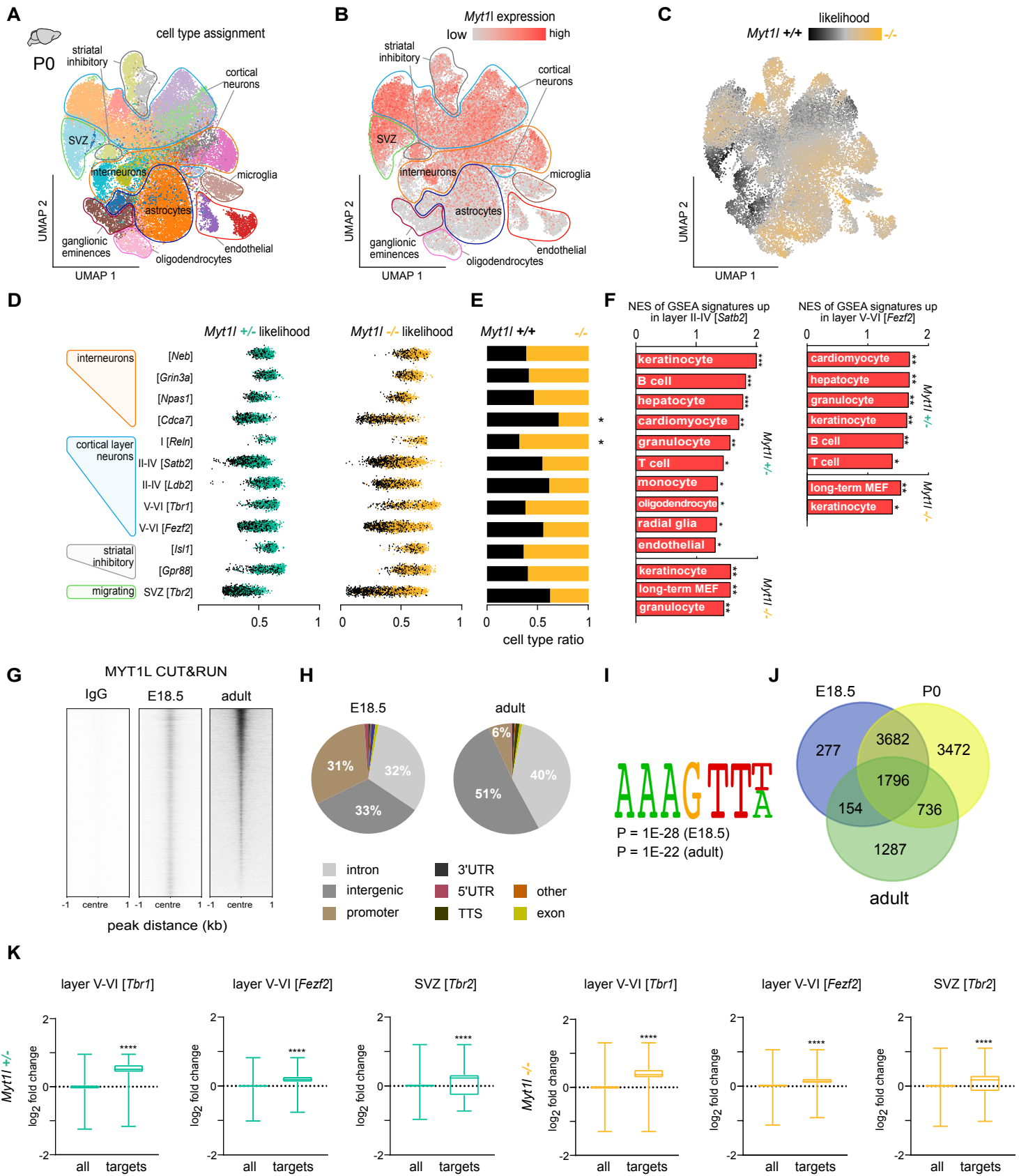

Supplement: Supplementary file 3 — Supplementary Figure 2 [file 41380_2023_1959_MOESM3_ESM.pdf]

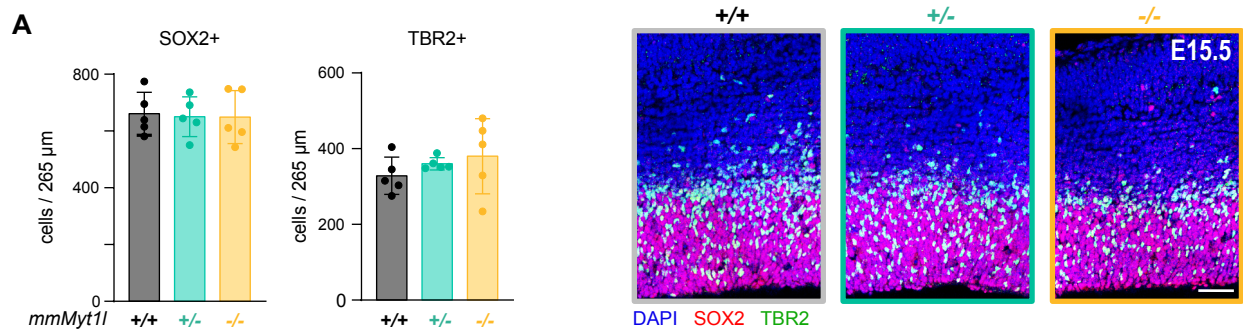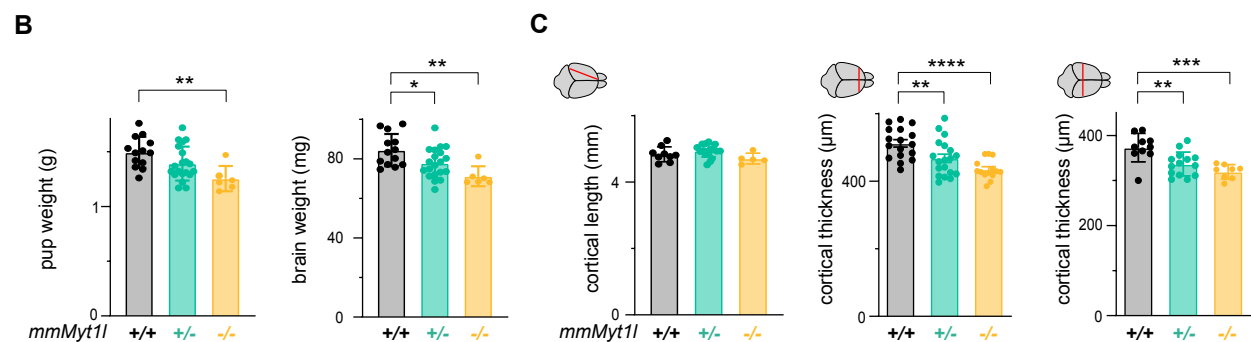

Supplement: Supplementary file 4 — Supplementary Figure 3 [file 41380_2023_1959_MOESM4_ESM.pdf]

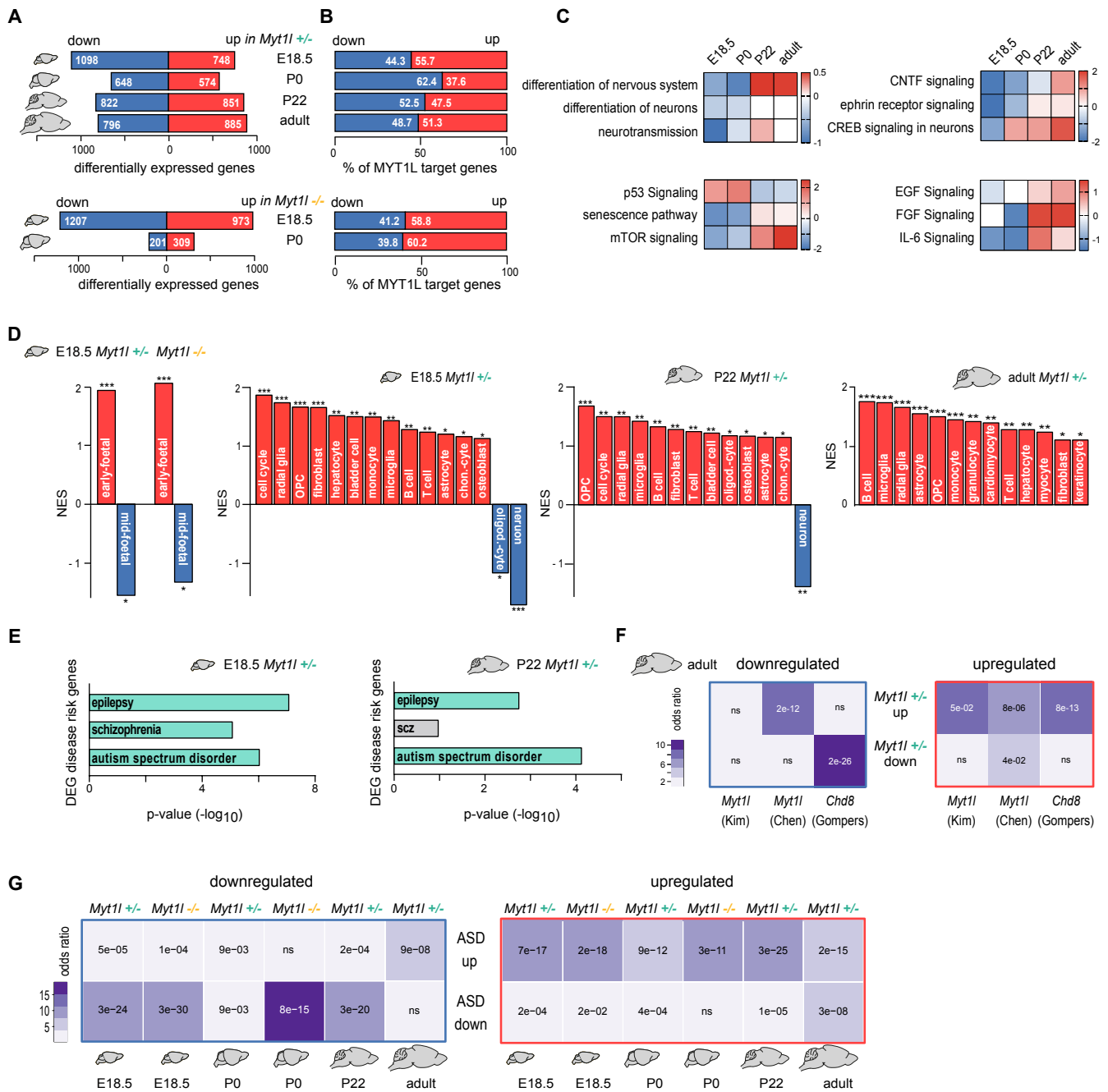

Supplement: Supplementary file 5 — Supplementary Figure 4 [file 41380_2023_1959_MOESM5_ESM.pdf]

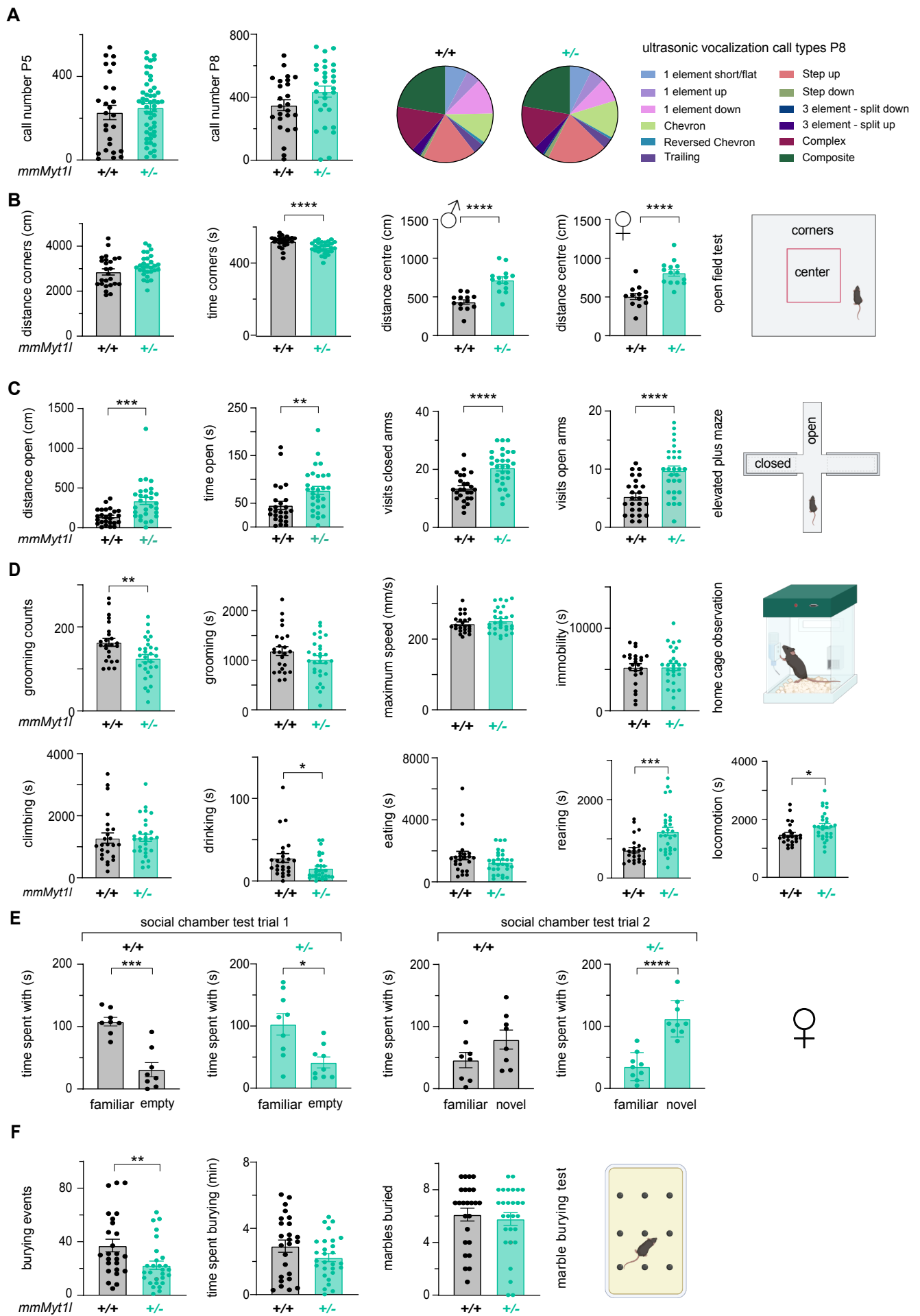

Supplement: Supplementary file 6 — Supplementary Figure 5 [file 41380_2023_1959_MOESM6_ESM.pdf]

A

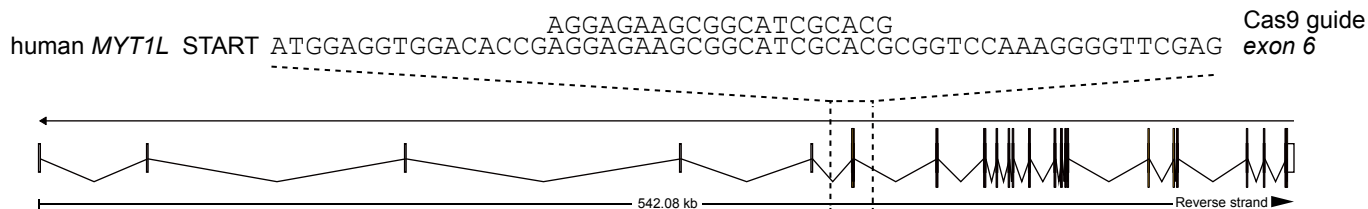

B

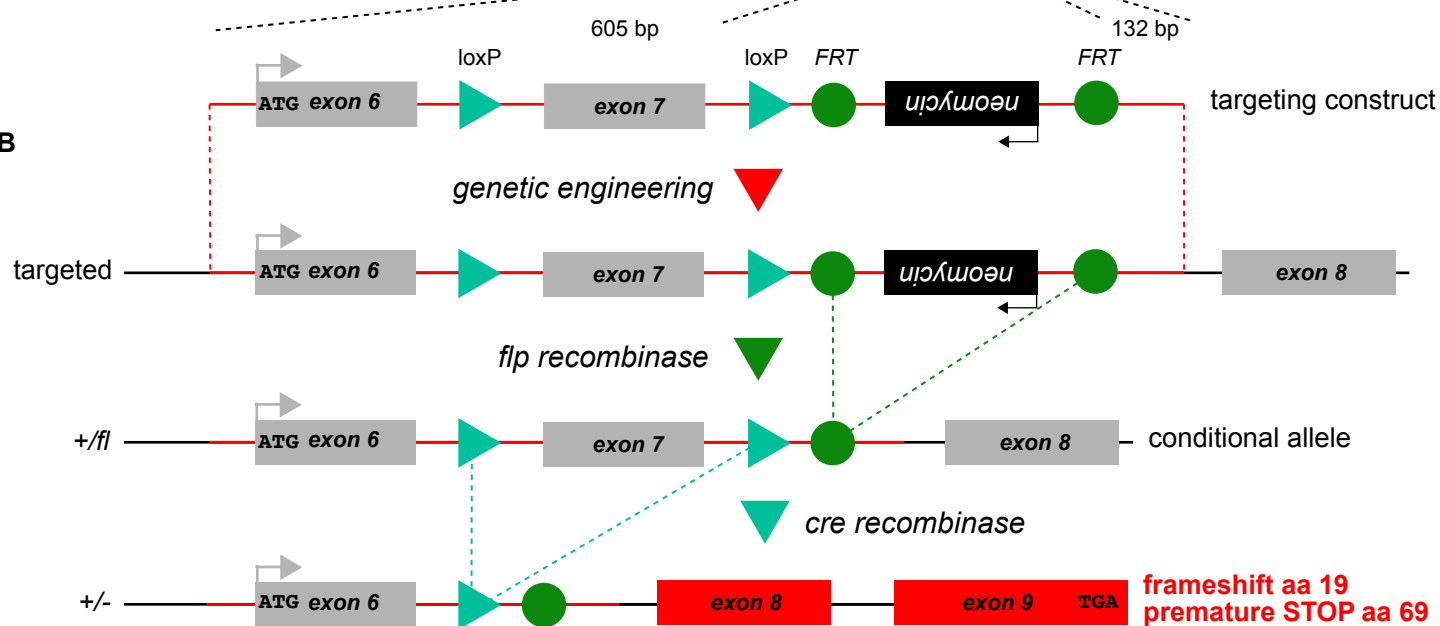

C

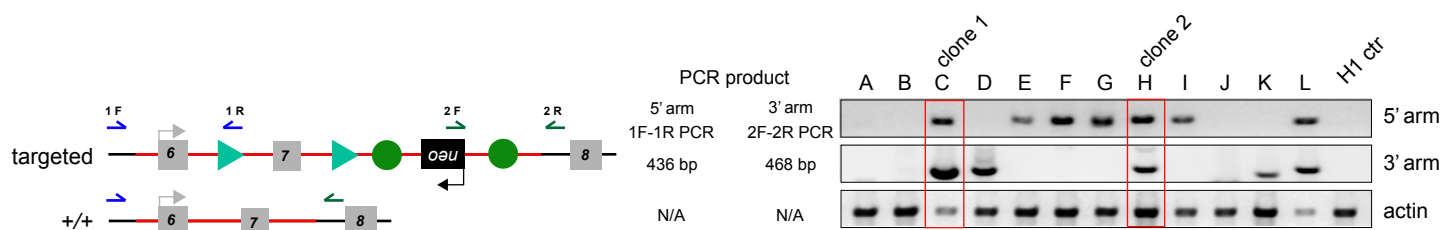

D

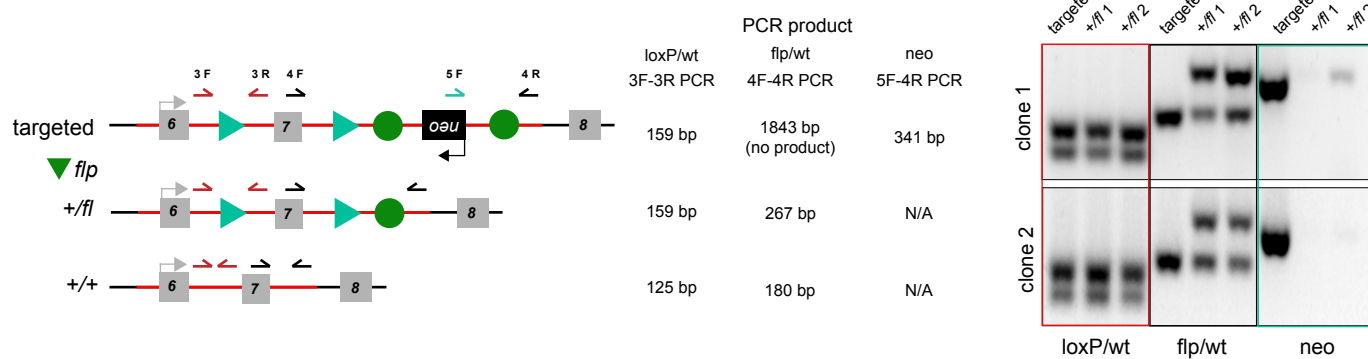

E

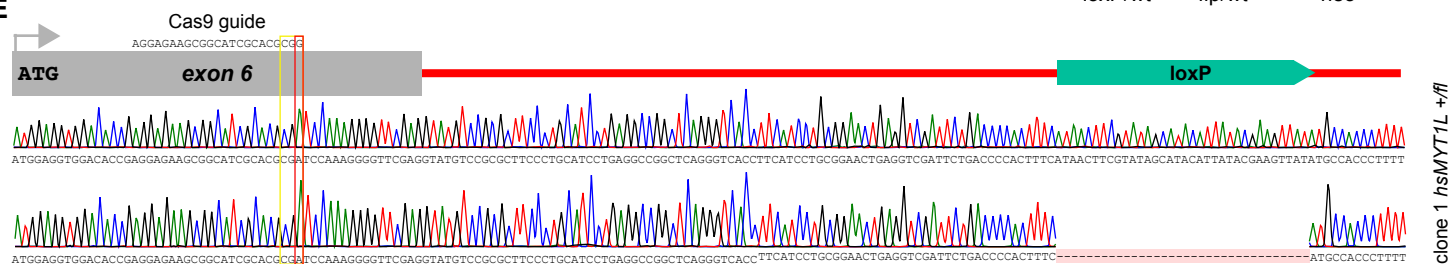

F

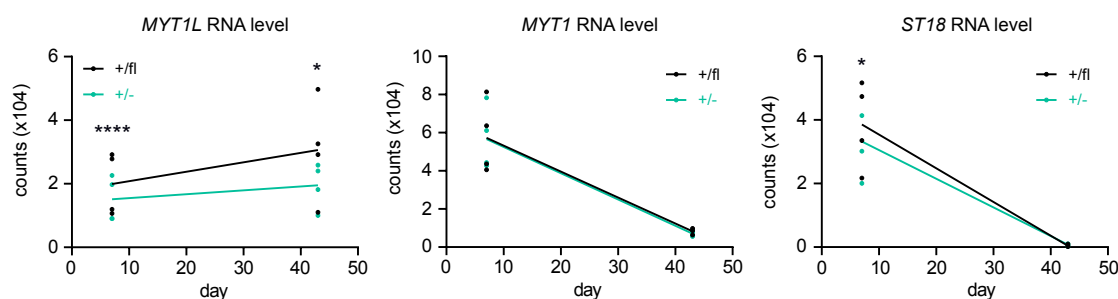

G

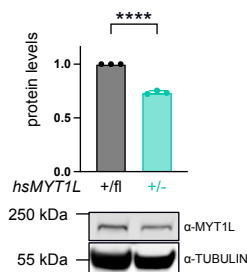

Supplement: Supplementary file 7 — Supplementary Figure 6 [file 41380_2023_1959_MOESM7_ESM.pdf]

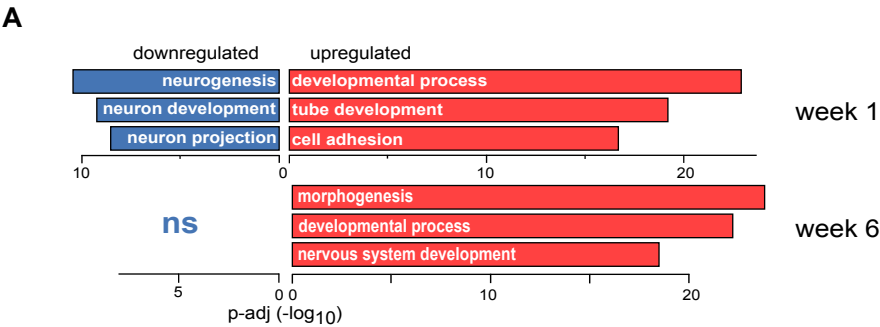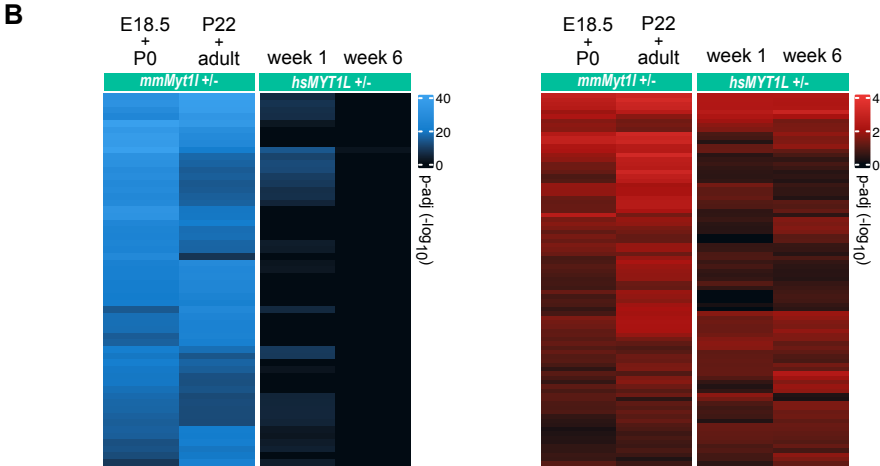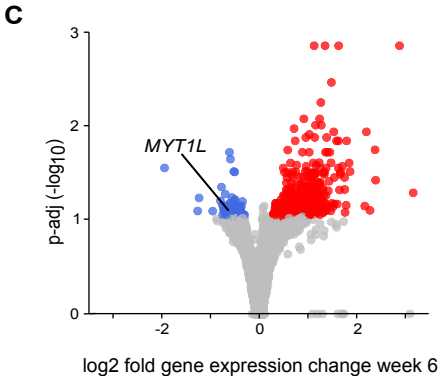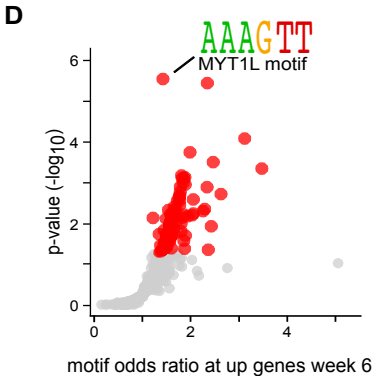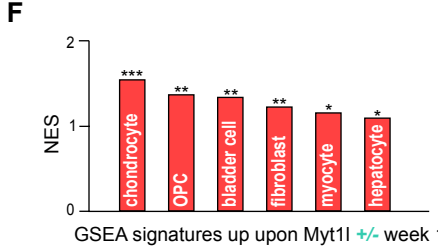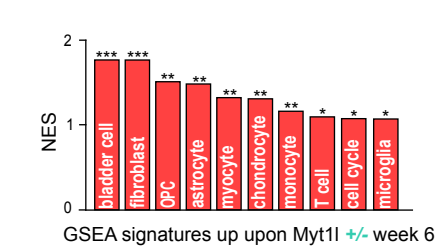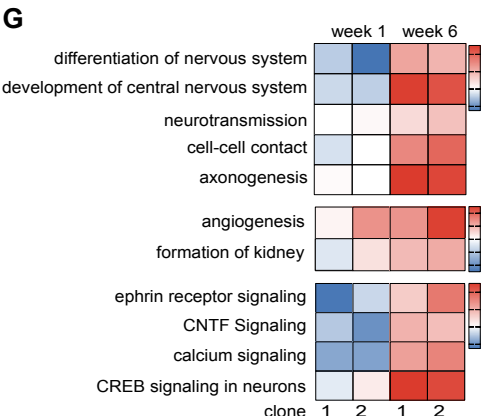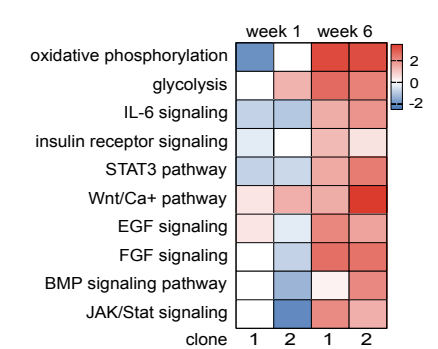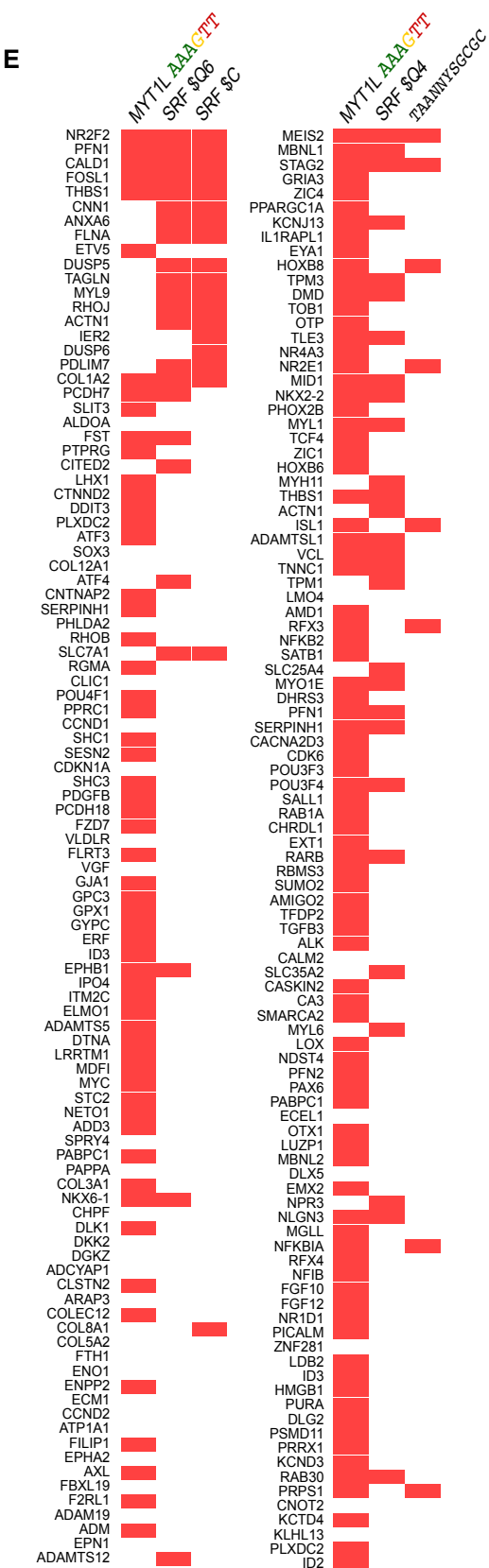

Supplement: Supplementary file 8 — Supplementary Figure 7 [file 41380_2023_1959_MOESM8_ESM.pdf]

**A**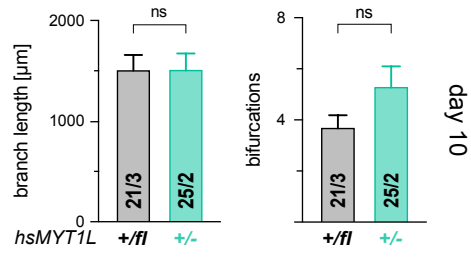**C**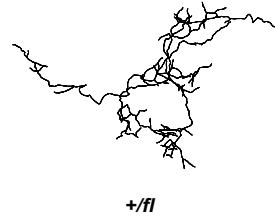**B**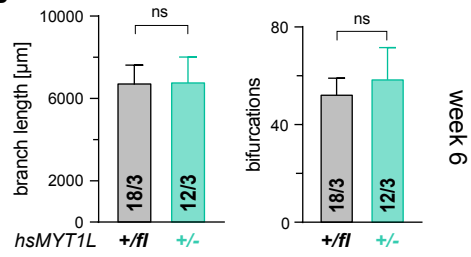

week 6

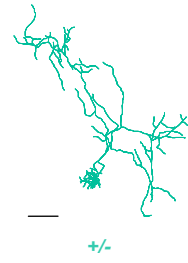

Supplement: Supplementary file 9 — Supplementary Figure 8 [file 41380_2023_1959_MOESM9_ESM.pdf]

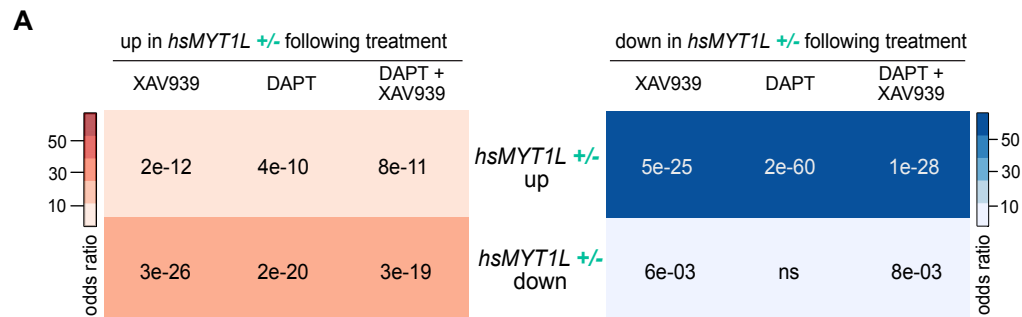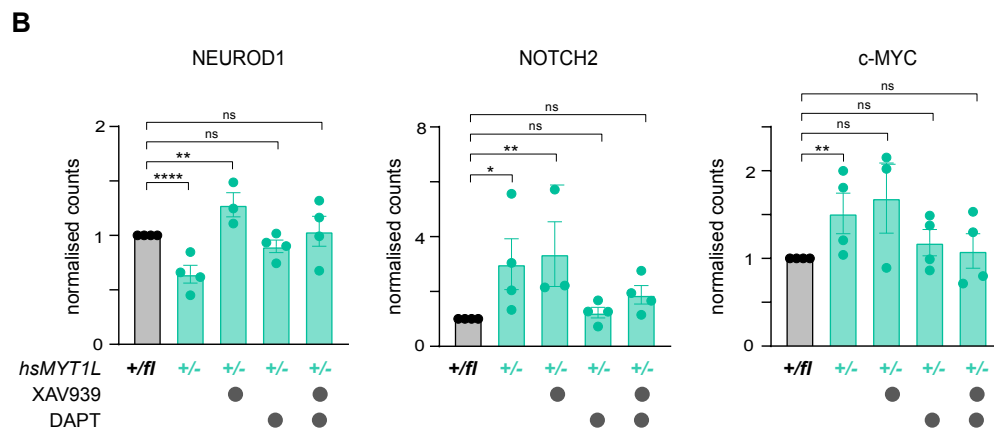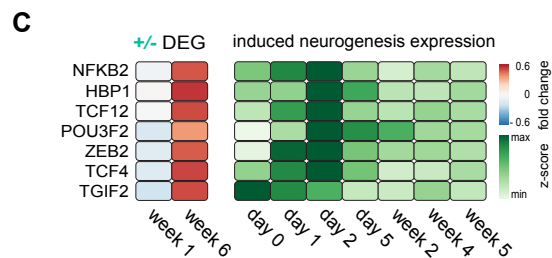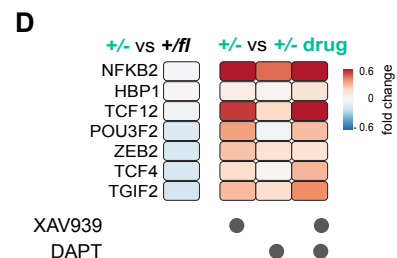

Supplement: Supplementary file 10 — Supplementary Figure 9 [file 41380_2023_1959_MOESM10_ESM.pdf]

**A**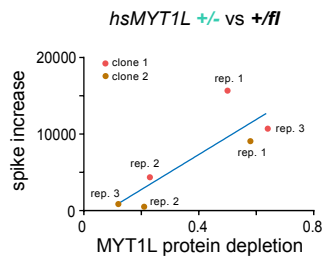**B**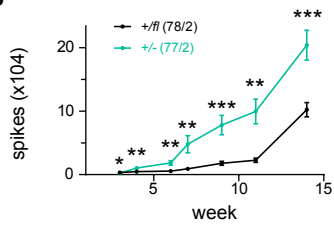**C**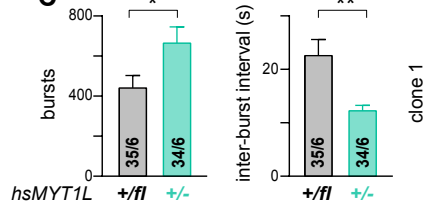**D**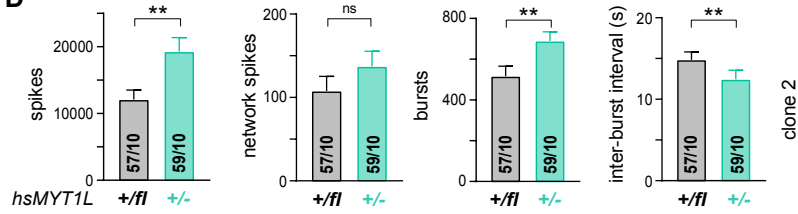

Supplement: Supplementary file 11 — Supplementary Figure 10 [file 41380_2023_1959_MOESM11_ESM.pdf]

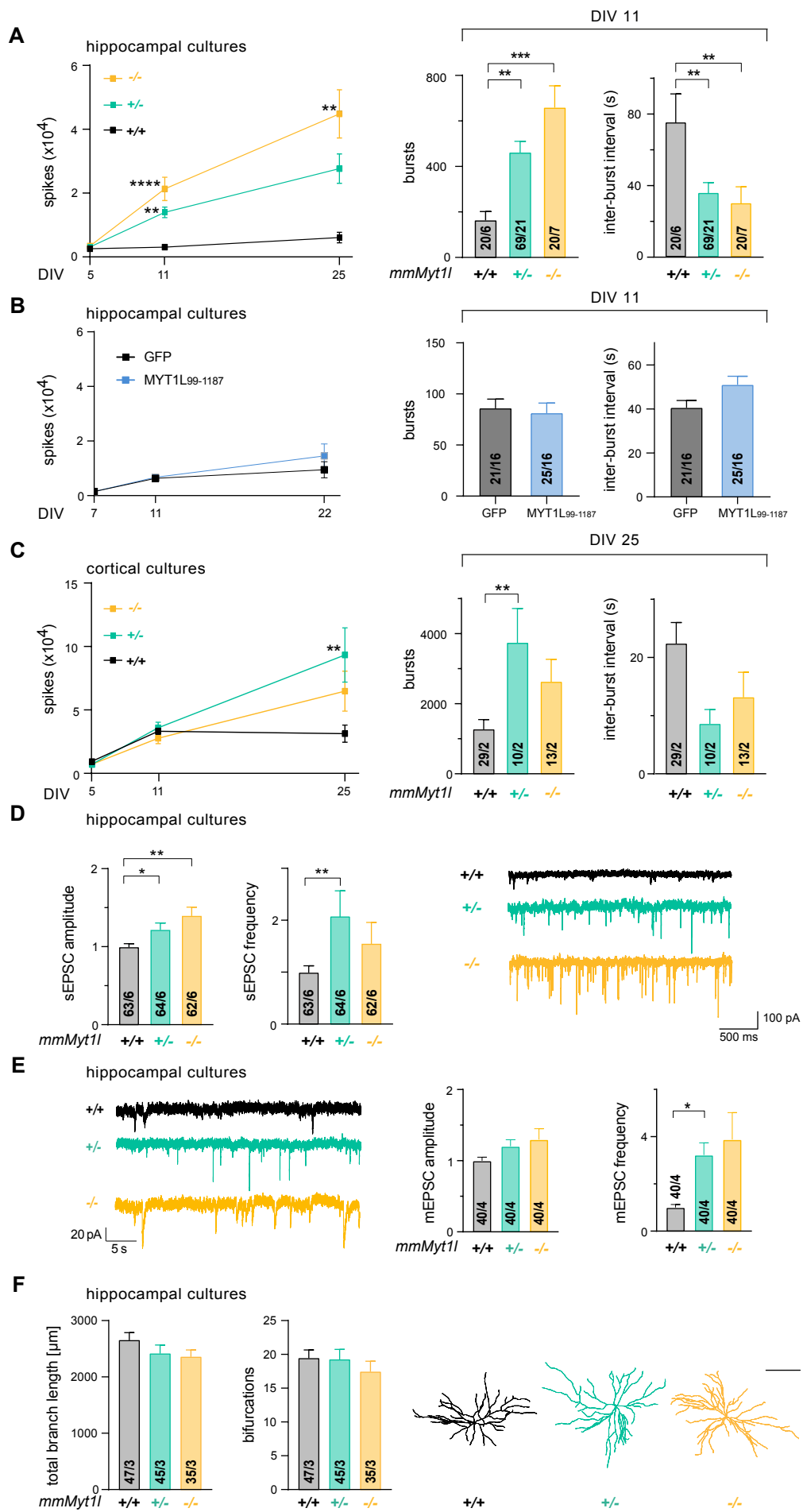

Supplement: Supplementary file 12 — Supplementary Figure 11 [file 41380_2023_1959_MOESM12_ESM.pdf]

**A**

acute hippocampal brain slices

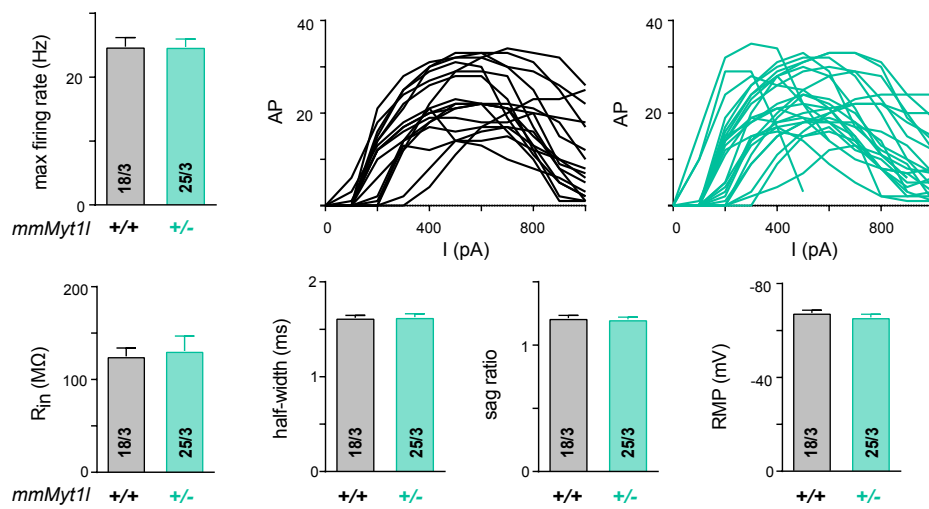**B**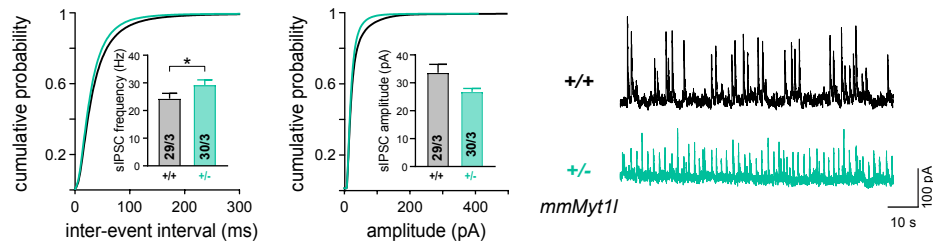

Supplement: Supplementary file 13 — Supplementary Figure 12 [file 41380_2023_1959_MOESM13_ESM.pdf]

**A**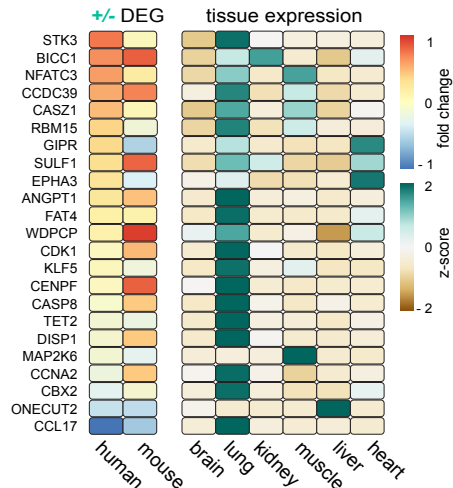**B**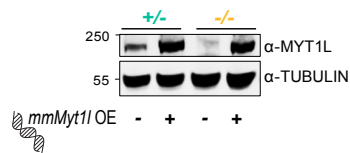**C**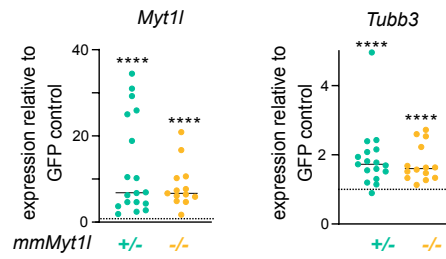**D**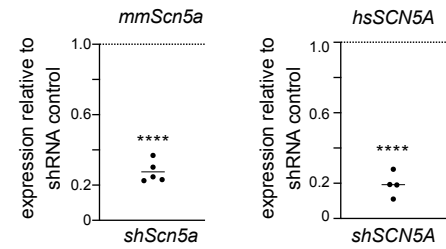**E**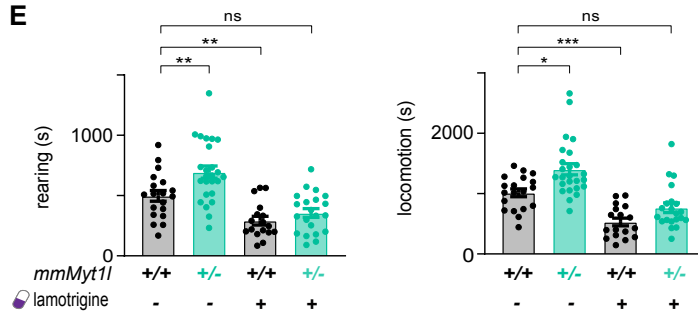

home cage observation

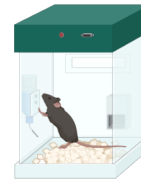

Supplement: Supplementary file 14 — Supplementary Figure 13 [file 41380_2023_1959_MOESM14_ESM.pdf]
